# ALASCA: An R package for longitudinal and cross-sectional analysis of multivariate data by ASCA-based methods

**DOI:** 10.3389/fmolb.2022.962431

**Published:** 2022-10-26

**Authors:** Anders Hagen Jarmund, Torfinn Støve Madssen, Guro F. Giskeødegård

**Affiliations:** ^1^ Department of Clinical and Molecular Medicine, Norwegian University of Science and Technology (NTNU), Trondheim, Norway; ^2^ Centre of Molecular Inflammation Research (CEMIR), NTNU, Trondheim, Norway; ^3^ Department of Circulation and Medical Imaging, NTNU, Trondheim, Norway; ^4^ K.G. Jebsen Center for Genetic Epidemiology, Department of Public Health and Nursing, NTNU, Trondheim, Norway

**Keywords:** R, omics analysis, statistical method, ASCA, longitudinal data analysis, multivariate analysis

## Abstract

The increasing availability of multivariate data within biomedical research calls for appropriate statistical methods that can describe and model complex relationships between variables. The extended ANOVA simultaneous component analysis (ASCA^+^) framework combines general linear models and principal component analysis (PCA) to decompose and visualize the separate effects of experimental factors. It has recently been demonstrated how linear mixed models can be included in the framework to analyze data from longitudinal experimental designs with repeated measurements (RM-ASCA^+^). The ALASCA package for R makes the ASCA^+^ framework accessible for general use and includes multiple methods for validation and visualization. The package is especially useful for longitudinal data and the ability to easily adjust for covariates is an important strength. This paper demonstrates how the ALASCA package can be applied to gain insights into multivariate data from interventional as well as observational designs. Publicly available data sets from four studies are used to demonstrate the methods available (proteomics, metabolomics, and transcriptomics).

## 1 Introduction

The increasing availability of high-dimensional data through omics-technologies can yield new insights into how intricate biological systems evolve and how they respond to various experimental conditions. However, there is a need for parallel development of novel statistical methods that can deal with the increased complexity of such data. The methods must be valid for multidimensional data sets, flexible for different experimental settings, as well as interpretable. Commonly used methods for multivariate data analysis, such as principal component analysis (PCA) and partial least squares (PLS) regression, are not able to fully account for more complex experimental designs. Multilevel PLS-DA, for instance, can only handle two time points, and adjusting for confounders can only be handled by subgroup analysis. One powerful approach for analysis of multivariate data is the ANOVA simultaneous component analysis (ASCA) framework that combines ANOVA with PCA (Smilde et al., 2005; Smilde et al., 2012). More recently, extended ASCA methods such as ASCA^+^ (Thiel et al., 2017), LiMM-PCA, and repeated measures ASCA^+^ (RM-ASCA^+^, Martin and Govaerts, 2020; Madssen et al., 2021) have emerged that combine general linear (mixed) models with PCA. In this way the flexibility of regression models are merged with the visualization of multivariate analysis, providing excellent interpretability by allowing to separate and display the complex multivariate patterns originating from different experimental factors. Despite these benefits, the availability of software implementations of ASCA^+^, and thus the use of the framework, has been limited.

In short, (RM-)ASCA^+^ comprises three steps: first, linear regression with or without random effects produces regression coefficients (*β*) which are summarized into a fixed effect parameter matrix (**B**, also including fixed intercepts). In the case of *K* measurements of *J* variables in *I* individuals, the linear mixed model based regression with *R* random effect coefficients (*γ*, including intercepts) and *p* fixed effect coefficients (*β*, including the intercept) can be written as

Y=XB+ZU+E, (1)

where **Y** is an *IK* × *J* response matrix, **X** is an *IK* × *p* design matrix, **B** is a *p* × *J* parameter matrix, **Z** is an *IK* × *R* design matrix for random effects, **U** is an *R* × *J* random parameters matrix, and **E** is an *IK* × *J* residual matrix. Equation 1 can also be written as


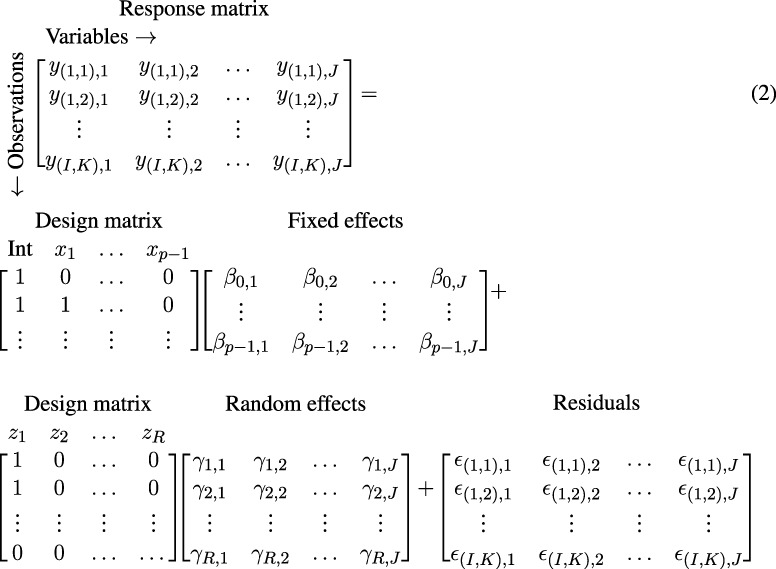


where the design matrices are filled with custom values for demonstration, *y*_(*i*,*k*),*j*_ is the *k*th measurement of variable *j* in individual *i*, and *ϵ*_(*i*,*k*),*j*_ the corresponding residuals. It will in many cases be sufficient to include a random intercept for participant. **ZU** is then simplified to an *IK* × *J* matrix with one intercept per individual per variable (*γ*_*r*,*j*_ → *γ*_*i*,*j*_), repeated for *K* rows. The subject-specific random intercepts (*γ*_*i*,*j*_) and the residuals (*ϵ*_(*i*,*k*),*j*_) are assumed to be normally distributed with mean zero and variations $\sigma_{u}^{2}$ and $\sigma_{e}^{2}$ , respectively. Ordinary ASCA^+^ represents the special case when no random effects are included. The second step in RM-ASCA^+^ is to decompose the **XB** matrix into effect matrices **M**_*h*_ representing specific parts of the regression model,

$$XB=M_{0}+\underset{h}{\sum }M_{h}.$$ (3)

Here, **M**_0_ represents the intercept and is typically of little interest. In ordinary ASCA, **M**_0_ usually represents the grand mean matrix, whereas in RM-ASCA^+^ it typically either represents the baseline mean of all, or one of the groups, depending on how the effects are coded in the model. The effects *h* reflect the statistical and experimental design (for examples, *see* Madssen et al., 2021). In the context of a longitudinal study, an effect matrix **M**_*T*_ would represent the effect of time, i.e., the change from baseline. If the study comprises multiple groups, additional effect matrices describing group differences (**M**_*G*_) and time-group interaction (**M**_*T*:*G*_) would be appropriate. Other covariates included in the regression model, such as gender or body mass index (BMI), would also require a separate effect matrix. The final step in RM-ASCA^+^ is to apply PCA to individual or combined effect matrices, depending on the research question, and extract scores and loadings. The resulting scores and loadings can then be plotted to visualize how variables are affected by the selected effects.

Providing an estimate of uncertainty and robustness is an important feature for all statistical techniques. There is a risk of overfitting when using (RM-)ASCA^+^, as (RM-) ASCA^+^ is a supervised method applied to labeled data (Bertinetto et al., 2020). To mitigate the risk of overfitting, the confidence of the estimated scores and loadings from (RM-)ASCA^+^, reflecting the effects of factors and possibly their interaction, should be tested. Most common are resampling methods such as bootstrap, jack-knife and permutation (Vis et al., 2007; Bertinetto et al., 2020). The latter involves random shuffling of the data labels before applying (RM-)ASCA^+^, often 1,000–10,000 times. As no systematic relationships should exist in the data when measurements are shuffled across experimental conditions, it establishes null-distributions for scores, loadings, or other metrics. A *p*-value can then be calculated by comparing the metric from the unaltered model to the null-distributions. While exact permutation tests exist for main effects, only approximate tests are available for interaction effects (Anderson and Braak, 2003; Bertinetto et al., 2020). In contrast to the permutation test, the bootstrap and jack-knife methods conserve the data labels. Here, the robustness of the metrics are tested by applying (RM-)ASCA^+^ to either a subset of the original data set, where a proportion of the participants are excluded (jack-knife), or a resampled data set, where individual participants are selected at random with replacement (bootstrap). When this is repeated in the order of 1,000–10,000 times, confidence intervals can be estimated for the scores and loadings by extracting upper and lower percentiles from the results of the resampled data sets. Multiple strategies exist for permutation testing (Anderson and Braak, 2003), and their suitability for RM-ASCA^+^ models with various designs is currently under investigation.

The Assorted Linear functions for ASCA (ALASCA) package for R has been developed to make the ASCA^+^ and RM-ASCA^+^ frameworks accessible for the general researcher. The package does not require advanced programming skills and is publicly available from the Github code repository (https://github.com/andjar/ALASCA). Although the ALASCA package supports both ASCA^+^ and RM-ASCA^+^ analysis, the main focus of this paper will be analysis of repeated measures of multivariate data with RM-ASCA^+^ due to the increasing need for flexible methods to deal with longitudinal experimental designs. The package utilizes well-known R syntax for defining regression models, offers options for predefined or custom scaling, includes multiple validation methods (jack-knifing and bootstrapping), and produces publication-ready figures. While the package is designed to be easy to use, it provides a wide range of customizable options available for advanced users. Further, the package includes several options for exporting the resulting models for archival, post-processing, external visualization, or sharing. Earlier versions of the ALASCA package has been used to reveal how serum cytokine levels change throughout pregnancy in healthy women (Jarmund et al., 2021) and in women with polycystic ovary syndrome (Stokkeland et al., 2022), and to show how the cytokine development is sensitive to maternal and fetal factors. The flexibility of the RM-ASCA^+^ framework was crucial for the combination of multiple cohorts and for making complex relationships available for interpretation. Since then, the package has been further developed for general use and includes new functions for validation and visualization.

In this paper, we demonstrate how the ALASCA package can be used to analyze various multivariate omics-data using RM-ASCA^+^. Three publicly available data sets are analyzed to illustrate each modeling step, including appropriate choice of scaling, model setup, and validation technique, and to demonstrate how the results can be easily visualized and interpreted. The data sets are diverse in terms of biological level (proteomics, metabolomics, transcriptomics) and experimental design (repeated measures within observational and randomized-controlled intervention studies). This practical and integrated approach will demonstrate the flexibility of the ALASCA package for data exploration and analysis.

### 1.1 Related works

Previous implementations of ASCA and ASCA-related methods exist for several common statistical software such as R and Matlab (Bertinetto et al., 2020). The first implementation of ASCA was published as Matlab scripts by Smilde et al. (2005). For R, the earliest implementations include ASCA-genes (Nueda et al., 2007, the scripts are no longer available) and the lmdme package (Fresno et al., 2014). Later options include MetStaT (removed from CRAN but available as archive https://cran.r-project.org/src/contrib/Archive/MetStaT/) for R and the PLS_toolbox and MetaboAnalyst (Xia et al., 2015) for Matlab (Bertinetto et al., 2020).

The multiblock package for R offers a comprehensive set of methods for multiblock analysis, including various ASCA-based methods such as LiMM-PCA, generalized ASCA, RM-ASCA^+^, and covariates ASCA (Liland, 2022; Smilde et al., 2022). A Matlab implementation of RM-ASCA^+^ has been published by Madssen et al. (2021), (scripts available at https://github.com/ntnu-mr-cancer/RM_ASCA). An extension of RM-ASCA^+^ has been proposed in the case of zero-inflated count data, namely the zero-inflated counts (ZIC)RM-ASCA^+^ by applying zero-inflated negative binomial mixed models, with code available for R (https://github.com/AukeHaver/ZICRM-ASCA_plus).

The ALASCA package offers several distinct features compared to existing implementations such as integrated scaling and validation, option to force equal baseline (important for randomized designs), supports both sum and contrast coding, precise yet simple specification of effect matrices, and diverse options for visualization.

## 2 Materials and methods

### 2.1 Package overview

**FIGURE 1:**
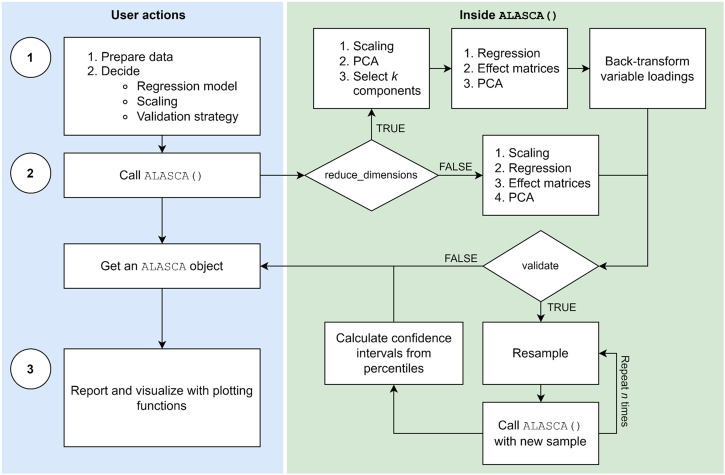
The typical workflow in the ALASCA package involves three stages: (1) Preparation, (2) execution, and (3) visualization. When the user has prepared the data and decided regression model, scaling, and validation strategy, the ALASCA() function is called. The ALASCA() function will then scale the data, perform regression analyses, apply principal component analysis (PCA) to the effect matrices, and extract loadings and scores. The option reduce_dimensions = TRUE will use PCA to reduce the number of variables to *k*, and loadings are automatically transformed back to the original variable space. Validation is performed if validate = TRUE is specified. The validation consists of performing (RM-)ASCA^+^ on *n* resampled data sets, and using percentiles for loadings and scores for confidence intervals. When the model is constructed, the user can visualize and report results in various ways.

The main functions of the ALASCA package are described in Table 1 and a typical work flow is illustrated in Figure 1. The ALASCA() function is used to define the regression model, scaling, and validation strategy. The resulting ALASCA object can then be visualized in several ways.

**TABLE 1:** Important functions in the ALASCA package.

Function	Description
ALASCA()	Initialize and create the ALASCA model
flip()	Invert the signs of scores and loadings
plot(…, type = “effect”)	Plot scores and loadings from a model
plot(…, type = “prediction”)	Plot marginal means from the underlying regression models
plot(…, type = “validation”)	Plot score and loading for all validation runs
plot(…, type = “histogram”)	Plot score and loading for all validation runs as histograms
plot(…, type = “residuals”)	Plot regression residuals
plot(…, type = “covars”)	Plot regression coefficients of covariates
plot(…, type = “2D”)	Plot the main results of the model
plot(…, type = “participants”)	Plot measurements from individual participants
summary()	Returns key information about the model
get_scores()	Returns the scores of the model
get_loadings()	Returns the loadings of the model
get_covars()	Returns additional regression coefficients
get_predictions()	Returns marginal means from the model

The ALASCA() function accepts a range of arguments related to the regression model and validation (Table 2). Recommended arguments for various study designs and research questions are demonstrated in the examples below. ALASCA will fit linear mixed models if the regression formula contains terms with | such as (1|ID) (i.e., random effects) and ordinary linear regression models otherwise. Regression coefficients are estimated with one of three algorithms, depending on the specific model to be fitted, namely, the Rfast package (Papadakis et al., 2021), the lme4 package (Bates et al., 2015), or base lm (R Core Team, 2020). Coefficients are estimated by Rfast as default due to performance, but Rfast has some limitations on which regression models can be fitted. Therefore, lme4 and lm can be used as alternatives when more complex regression models are used. The two latter can be applied by specifying use_Rfast = FALSE and will also produce *p*-values and additional information such as *R* ^2^ for each regression model. When lme4 is used, *p*-values are calculated with Satterthwaite’s degrees of freedom method with the lmerTest package (Kuznetsova et al., 2017). The data.table package is extensively used to improve performance by doing data manipulation by reference and other optimizations (Dowle and Srinivasan, 2021). ALASCA objects are also manipulated by reference with help of the R6 package (Chang, 2021). Traditionally in R, functions will not modify variables in place but requires that variables are reassigned. ALASCA objects, however, can be modified without re-assignment. For instance, both flip(model) and model <- flip(model) will modify the model object.

**TABLE 2:** Important arguments for the ALASCA() function. A full list of arguments can be shown in R using ?ALASCA::ALASCA().

Function	Default	Description
df	—	Data frame containing the data set to be analyzed
formula	—	Regression formula
scale_function	“sdall”	Function to scale data. See description of possible defaults in the text
separate_effects	FALSE	When TRUE, separate effect terms
equal_baseline	FALSE	When TRUE, remove interaction at baseline
validate	FALSE	When TRUE, validate the model
reduce_dimensions	FALSE	When TRUE, use principal component analysis to reduce the number of variables
wide	FALSE	Set to TRUE if data are provided in wide format
stratification_column	NULL	Name of the column to be used for stratification during validation. By default, use group or first the effect term
validation_method	“bootstrap”	Set to “jack-knife” to use jack-knife resampling for validation
n_validation_runs	1000	Number of validation runs
save	FALSE	When TRUE, automatically save the model and subsequent plots
limitsCI	c(0.025, 0.975)	Lower and upper percentiles for confidence intervals

Currently, model validation can be performed with cluster bootstrap or jack-knife, both with stratification. During validation, the ALASCA() function will call itself using a modified data set n_validation_runs times (Figure 1). The default is 1,000 runs. If cluster bootstrap is selected (default), each participant is replaced by a randomly selected participant from the same stratification group, with replacement, and all measurements from the sampled participant are added to the modified data set. If jack-knife is chosen, the stratification groups are iterated and one out of *q* (defaults to *q* = 7) participants are excluded at random from the iteration. By default, any column named group in the data set df will be used for stratification, i.e., the relative group sizes are kept during validation. Alternatively, another column in df can be specified for stratification as stratification_column. If there is no group column and stratification_column is not specified, the first effect term will be used for stratification. Loadings from the validation runs are rotated towards loadings from the initial run using procrustes rotation, and the rotation matrix is applied to the scores from the validation run as well. As the sign of loadings and scores in PCA is arbitrarily defined, ALASCA() will test whether changing the signs of each principal component (PC) improves the fit of the scores from validation runs and the initial run, and choose the signs minimizing the summed distance of the scores. Only PCs explaining more than 5% variance are used for rotation. Finally, 95% confidence intervals (CIs) are calculated for scores and loadings by selecting the 2.5% and 97.5% percentiles from the validation runs.

Visualizations are made within the popular ggplot2 framework (Wickham, 2016; Kassambara, 2020; Slowikowski, 2021). The default color palette for figures is the viridis palette which is designed to be readable and perceptually uniform despite gray scale printing and the most common forms of color blindness (Wickham and Seidel, 2020; Garnier et al., 2021). Custom ggplot2 themes can be used by specifying plot.my_theme. If save = TRUE was used during initialization of the model, the plot() function will automatically save all plots that are produced.

For megavariate data sets, the large number of measured variables makes individual regression too time consuming for validation with sufficient numbers of iterations. If reduce_dimensions = TRUE, ALASCA() will perform an initial PCA on the measurements, prior to regression, so that the original variables are replaced by PCs (Figure 1), similar as for Limm-PCA (Martin and Govaerts, 2020). The number of PCs kept from the initial PCA is selected so that 95% of the variance in the measurements is explained. The limit can be changed by specifying reduce_dimensions.limit. Additionally, one can prevent ALASCA from running out of memory by saving results from the validation runs directly to a duckdb or sqlite3 database instead of keeping all the results in memory with save_to_disk = TRUE (R Special Interest Group on Databases et al., 2021; Müller et al., 2021; Mühleisen and Raasveldt, 2022).

Logging of important events, such as estimated time for validation or error messages, is performed with the log4r package and written to file by default (White and Jacobs, 2021).

### 2.2 Installation and data preparation

The ALASCA package is freely available at the Github code repository and can be installed in R with the following commands:

install.packages(“devtools”)

devtools:install_github(“andjar/ALASCA”, ref = “main”)

Version 1.0.0 of ALASCA was used for this paper. The code to reproduce all results in this paper, including data preparation and figures, can be found in the supplementary materials, and simplified function calls are given below. The full code in the supplementary materials utilizes additional packages such as here and reshape2.

The ALASCA() function requires at minimum a data frame or data table df and a regression formula. Generally, data can be organized in two formats (Supplementary Figure S1): long (all measured variables have separate rows) or wide (all observations have separate rows, with the different variables as separate columns). If data are provided to ALASCA() in long format with one row for each measured variable (Supplementary Figure S1A and examples 1 and 3 below), the variable names (i.e., the measured variables) must be in a column named variable. If wide format is used (one row per measured sample, with variables as separate columns, Supplementary Figure S1B and example 2 below), wide = TRUE must be provided to ALASCA() and all columns not mentioned in the formula or being specified otherwise (Table 2) will be treated as columns containing measurements of interest. At least two other columns are required, regardless of format: One column must contain an identifier for the experimental unit, typically the study numbers of the participants. By default, this column is either derived from the random intercept in the formula or, in case there are no or multiple random intercepts in the formula, it is assumed to be named ID. If another column is to be used, it must be specified as participant_column. Secondly, one column must contain the first effect of interest and will be used to label the *x*-axis in subsequent score plots. By default, this is assumed to be the first term in the formula. If another column is to be used, it must be specified as x_column. General data preparation is demonstrated in the supplementary files. For example, the function call

ALASCA(

df,

formula = value ∼ v1 + v2 + (1|ID),

validate = TRUE)

will assume that the provided data (df) is organized in long format (Supplementary Figure S1A) and includes the columns variable, value, v1, v2, and ID (random intercept). The regression formula value ~ v1 + v2 + (1|ID) corresponds to a model with value as outcome, ID as random intercept, and v1 and v2 as main effect terms. Bootstrap validation will also be applied as validate = TRUE with 1,000 iterations (default). If df contains a column called group, the observations will be stratified by group during bootstrapping, otherwise they are statified by v1. Since scaling has not been specified (*see* below), the outcome data will be scaled by the default method (i.e., division by the standard deviation, by variable).

The effects of interest can be specified (e.g., effects = c(“v1”, “v1:v2”) where v1, v2, … are terms in the regression formula) or inferred by ALASCA. In the latter case, the first formula term is assumed to be of interest. Next, ALASCA will look for an interaction term, and, if it exists, include the interaction and second main effect. For example, if the formula is value∼v1*v2 + v3 + (1|ID), ALASCA will assume that v1, v2, and v1:v2 (interaction) are all of interest. How they are combined depends on separate_effects. If separate_effects = FALSE (default), only one combined effect is extracted (i.e., v1*v2 or v1+v2+v1:v2). If separate_effects = TRUE, two separate effect matrices will be produced: v1 and v2+v1:v2. ALASCA will explicitly state which effects that are assessed when ran.

Columns representing effects of interest, typically the time and group columns, are expected to contain factors, i.e., categorical data with ordered levels. For example, df$group <- factor(df$group) will convert the group column to factors with the factor levels ordered alphabetically. The first levels of time and group are used as baseline or reference group. Level order can be specified explicitly, factor(…, levels = c(“Male,” “Female”)), or by specifying just the reference, relevel(…, ref = “Male”).

The data should not be normalized or scaled as part of the preparation. Instead, a scaling function must be specified and provided to the ALASCA() function. This prevents data leak during validation where a subset of the data set is used to determine scaling factors that are independently applied to the remaining data for validation. Four predefined options are currently available (Timmerman et al., 2015):

• scale_function = “sdall” will divide the value column by the standard deviation of all samples, by variable:

$${\hat{y}}_{\left(\cdot ,\cdot \right),j}=y_{\left(\cdot ,\cdot \right),j}/\text{SD}\left(y_{\left(\cdot ,\cdot \right),j}\right)$$

• scale_function = “sdt1” will divide the value column by the standard deviation of all baseline samples, by variable:

$${\hat{y}}_{\left(\cdot ,\cdot \right),j}=y_{\left(\cdot ,\cdot \right),j}/\text{SD}\left(y_{\left(\cdot ,k\right),j}\right),\;\;\;k=1$$

• scale_function = “sdref” will divide the value column by the standard deviation of all samples in the reference group, by variable:

$${\hat{y}}_{\left(\cdot ,\cdot \right),j}=y_{\left(\cdot ,\cdot \right),j}/\text{SD}\left(y_{\left(i,\cdot \right),j}\right),\;\;i\in \text{Reference group}$$

• scale_function = “sdreft1” will divide the value column by the standard deviation of all baseline samples in the reference group, by variable:

$${\hat{y}}_{\left(\cdot ,\cdot \right),j}=y_{\left(\cdot ,\cdot \right),j}/\text{SD}\left(y_{\left(i,k\right),j}\right),\;\;i\in \text{Reference group},\;\;\;k=1$$

where SD refers to the standard deviation, ${\hat{y}}_{\left(i,k\right),j}$ is the scaled and *y*_(*i*,*k*),*j*_ the raw value of variable *j* for individual *i* at time point *k* (*see* Eq. 2). Mean centering is by default performed before scaling. In addition, a custom scaling function can be provided. The scaling function should have the data frame as argument and return a data frame with scaled values:

scale_function <- function(df){

... # Scale the value column

return(df) }

### 2.3 Example 1: Observational design with repeated measurements

To illustrate the analysis of longitudinal, observational data, we use two publicly available proteomics data sets (Erez et al., 2017; Tarca et al., 2019) to approach the following research questions:

1. How does the plasma proteome develop throughout normal pregnancy?
2. How does smoking affect the plasma proteome development throughout normal pregnancy, when accounting for body mass index (BMI)?
3. Does the plasma proteome of pregnancies that are later complicated by early- or late-onset preeclampsia follow distinct developmental trajectories?

#### 2.3.1 Materials

The two data sets contain repeated measurements of 1,125 plasma proteins from pregnant women, and share the same control group (*n* = 90 women). The first study, by Tarca et al. (2019), focused on early-onset preeclampsia (*n* = 33 women), whereas the second study, by Erez et al. (2017), investigated late-onset preeclampsia (*n* = 76 women). BMI, smoking status, age, and parity were available for controls and early-onset preeclampsia cases only.

For the two first analyses, we selected control cases to visualize the normal plasma proteome development throughout pregnancy. To utilize as many serum samples as possible, the control samples were divided into five time intervals: first trimester ( $\leq 13^{+6}$ weeks, *n* = 76), early second trimester (14^+0^–21^+6^ weeks, *n* = 87), late second trimester (22^+0^–27^+6^ weeks, *n* = 43), early third trimester (28^+0^–33^+6^ weeks, *n* = 40), and late third trimester ( $\geq 34^{+0}$ weeks, *n* = 32). Only the first sample from each participant at each time interval was included.

For the second analysis, the data from the previous example are reused as BMI and smoking status were available for the all healthy women. Smoking was coded as a factor in the group column with non-smokers acting as reference. Pre-pregnancy BMI was included as a continuous covariate as BMI is a potential confounder in the analysis.

For the third analysis the data sets from Erez et al. (2017) and Tarca et al. (2019) were merged to assess whether the plasma proteome of EO- and LO-preeclamptic pregnancies developed along distinct trajectories. The two data sets shared the same control group. Since women who developed EO-PE did not deliver plasma samples in late pregnancy, we restricted the analysis to samples collected before week 32^+0^. The remaining plasma samples were divided by gestational age into four time intervals: before week 14^+0^ ( $\leq 13^{+6}$ weeks), week 14–21 (14^+0^–20^+6^), week 21–28 (21^+0^–27^+6^), and week 28–32 (28^+0^–31^+6^).

### 2.4 Example 2: Randomized intervention with repeated measurements

To demonstrate how data from randomized intervention studies with repeated measurements can be analyzed with RM-ASCA^+^, we investigated a publicly available metabolomics data set from Euceda et al. (2017). In this data set, we aimed to assess the following research questions:

1. How is the metabolomic response in breast cancer affected by adding the drug bevacizumab to standard neoadjuvant chemotherapy?
2. How does the metabolomic response in breast cancer differ between responders and non-responders receiving neoadjuvant cheomtehrapy with or without bevacizumab?

Whereas Example 1 focused on the interpretation of models, this example will review scaling and validation strategies.

#### 2.4.1 Materials

The publicly available metabolomics data set from Euceda et al. (2017) contains measurements of 16 metabolites from 270 tumor biopsies from 122 patients randomized to either bevacizumab + chemotherapy (*n* = 60) or chemotherapy alone (*n* = 62). Biopsies were taken before treatment (*T*_1_), at 12 weeks into treatement (*T*_2_), and at tumor removal at 24 weeks (*T*_3_) and profiled with high resolution magic angle spinning MR spectroscopy (HR MAS MR). In total, 46 participants provided three biopsies, 21 in the chemotherapy group and 25 in the bevacizumab group. By time point, 105 (50% later received bevacizumab), 78 (47% receiving bevacizumab), and 87 (55% receiving bevacizumab) biopsies were available at *T*_1_, *T*_2_, and *T*_3_, respectively. Madssen et al. (2021) used this data set in the original description of RM-ASCA^+^ and their results are reproduced and further explored here using the ALASCA package.

For the second analysis, participants were classified as responders (*n* = 44) or non-responders (*n* = 78) on basis of tumor size at surgery (*T*_3_). In the chemotherapy group, there were 20 responders and 42 non-responders, and the corresponding numbers for the bevacizumab group were 24 and 36, respectively.

### 2.5 Example 3: Megavariate data

This example introduces dimension reduction which makes analysis of megavariate data sets manageable. A publicly available transcriptomics data set by Skaug et al. (2021) was analyzed to answer the following research questions:

1. Does skin gene expression differ between patients with systemic sclerosis (SSc) and healthy controls?
2. Does longitudinal skin gene expression differ between patients with limited and diffuse SSc?

#### 2.5.1 Materials

Skaug et al. (2021) collected forearm skin biopsies from 113 unique patients with limited (*n* = 43) or diffuse (*n* = 70) SSc and 44 matched healthy controls. Two additional biopsies were subsequently collected from a subset of the patients. A fourth biopsy was excluded due to the low sample size (*n* = 1). Gene expression was measured by RNA sequencing and microarrays. Variables with more than 10% missing values were excluded (1,065 genes), and the remaining missing values were replaced by half of the lowest measured value for the corresponding variable. To avoid duplicated gene names, “(d)” was added to the gene name when multiple probes assessed the same genes. In sum, 26,910 genes were kept for analysis.

## 3 Results and discussion

### 3.1 Example 1: Observational design with repeated measurements

#### 3.1.1 How does the plasma proteome develop throughout normal pregnancy?

Longitudinal plasma samples were collected from 90 healthy pregnancies and analyzed for 1,125 proteins. A possible model to assess normal proteome development throughout pregnancy involves a main effect for time (*k*) and a random intercept for each participant *i*. In R, this model can be specified as value∼time + (1|ID), where value is outcome, time the predictor, and ID the random intercepts. Since the first time point acts as baseline, protein levels were scaled by the standard deviation of the baseline samples by setting scale_function = “sdt1”. The RM-ASCA^+^ model was then initialized as

mod <- ALASCA(

df = df,

formula = value ∼ time + (1|ID),

scale_function = “sdt1”,

validate = TRUE

)

The corresponding design matrix is shown in Supplementary Table S1.

RM-ASCA^+^ extracted two general patterns of change as represented by the first (PC1) and second (PC2) principal component, explaining 87% and 9%, respectively, of the variability in the data set (Supplementary Figure S2). Each component is associated with positive and negative loadings describing how each plasma protein is related to the corresponding PC. Proteins with positive loadings have higher concentration in time points with higher score values, and *vice versa* for proteins with negative loading values.

**FIGURE 2:**
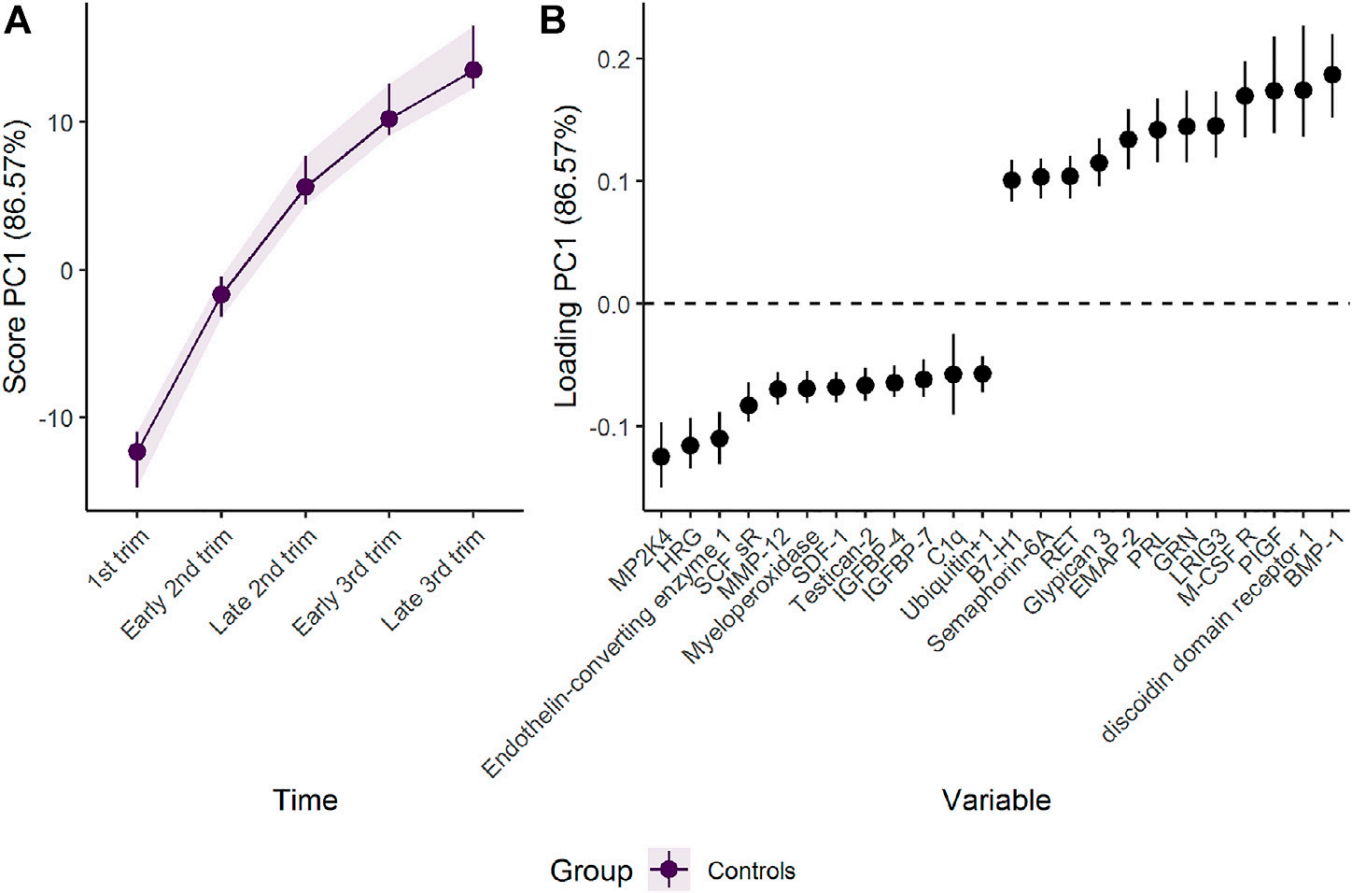
Time development of the plasma proteome throughout pregnancy as **(A)** scores and **(B)** loadings. The plasma level of proteins with high loading is increasing when the scores increase and *vice versa*. Only the 12 proteins with highest and lowest loadings, separated by the vertical dotted line, are shown due to the large number of assessed proteins.

**FIGURE 3:**
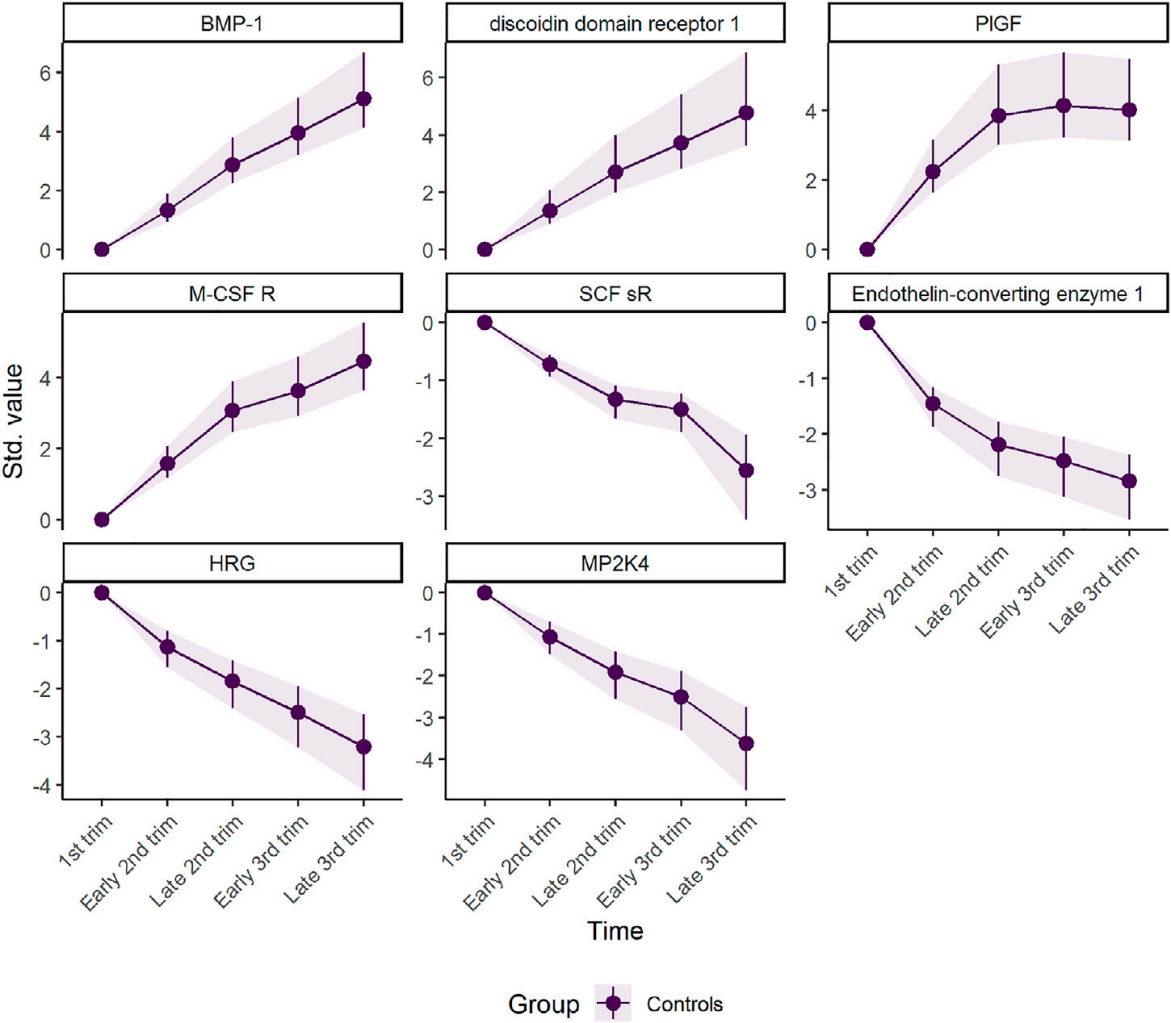
Marginal means for scaled protein concentration from linear mixed models. The intercept has been removed to highlight the robustness of development over time. The plot was made with the plot(…, type = “prediction”) function.

The first component represents a monotone increase (for positive loadings) or decrease (for negative loadings) in plasma level throughout pregnancy (Figure 2). The largest change takes place in the first and second trimester before stabilizing in the third trimester, as can be validated by assessing the underlying regression models (Figure 3). Bone morphogenetic protein 1 (BMP-1), epithelial discoidin domain-containing receptor 1 (EDDR1), and placenta growth factor (PlGF) showed the strongest positive loading on the first component, and therefore increase the most during the first trimesters. The increase of BMP1, EDDR1, and PlGF levels in plasma is clearly visible from the raw data itself (Supplementary Figure S3). In the opposite end, dual specificity mitogen-activated protein kinase kinase 4 (MAP2K4), histidine-rich glycoprotein (HRG), and endothelin-converting enzyme 1 (ECE1) showed the strongest negative loadings on the first component (Figure 2). This pattern is also evident from inspection of raw data (Supplementary Figure S3).

The second component represents a non-linear development with either peak (for positive loadings) or dip (for negative loadings) in the second trimester (Supplementary Figure S4). The first pattern is seen for proteins such as vascular endothelial growth factor A (VEGF-A), C1q and PAPPA-A. C1q did, however, show significant variability and had a CI for the loading that included zero. In contrast, the concentration of sialic acid-binding Ig-like lectin (siglec-) 6, Activin A, and IL-1 R4 showed a u-shaped dipping in the second trimester. These patterns are visible in the raw data as well (Supplementary Figure S5). Some variables had high loadings on both PC1 and PC2. Their trajectory is a combination of the two, as can be seen as flattening of the curve PlGF in the third trimester (Figure 3 and Supplementary Figure S3).

#### 3.1.2 How does smoking affect the plasma proteome development throughout normal pregnancy, when accounting for BMI?

The impact of smoking and pre-pregnancy BMI on plasma proteome development was examined in the same group of women as the analysis above (Section 3.1.1). Of the 90 pregnant women, 18 (20%) were smoking. Samples were collected from 76 (17% smoking), 87 (20% smoking), 43 (16% smoking), 40 (18% smoking), and 32 (19% smoking) women in the first trimester, early and late second trimester, and early and late third trimester, respectively. The BMI was 29 ± 7.8 and 28.1 ± 6.8 kg m^−2^ in the smoking and non-smoking group, respectively, and 28.3 ± 7.0 kg m^−2^ overall. The influence of BMI on the protein profile was assumed to be constant during pregnancy and thus there was no interaction with time in the regression model. In contrast, the effect of smoking was allowed to vary with time.

The regression formula was expanded to include a group term and time-group interaction: time*group is shorthand for time + group + time:group, where the two first terms represent the main effects of time and group, respectively, and the latter their interaction. Similarly, BMI was added as a covariate and the corresponding column kept as numerical values. The time and group effect matrices from Eq. 3 can be analyzed either separately or combined, so the model was ran twice, with separate_effects = TRUE, i.e., PCA is applied separately to **M**_*T*_ and **M**_*G*+*T*:*G*_, specified in the second run. The RM-ASCA^+^ models were initialized as

mod <- ALASCA(

df = df,

formula = value ∼ time*group + BMI + (1|ID),

scale_function = “sdt1”,

validate = TRUE

)

and

mod <- ALASCA(

df = df,

formula = value ∼ time*group + BMI + (1|ID),

separate_effects = TRUE,

scale_function = “sdt1”,

validate = TRUE

)

The corresponding design matrix is shown in Supplementary Table S2.

RM-ASCA^+^ offers two approaches to compare the time development of distinct groups of individuals. When the time and group effects are analyzed as a combined unit, i.e., the effect matrices for time, group, and time-group interaction in Eq. 3 are subjected to the same PCA, the resulting components will describe the common development of the groups. When the time and group effects are analyzed as separate units, i.e., the effect matrix for time is separated from the effect matrices for group and time-group interaction in Eq. 3 and analyzed separately by PCA, two sets of scores and loadings are extracted. The first set of scores and loadings describes the development of the reference group, whereas the second set describes how the other groups diverge from the reference group. The underlying regression models, as well as the resulting regression coefficients, are, however, the same for the two approaches as the matrices **X** and **B** in Eq. 3 remain unchanged.

**FIGURE 4:**
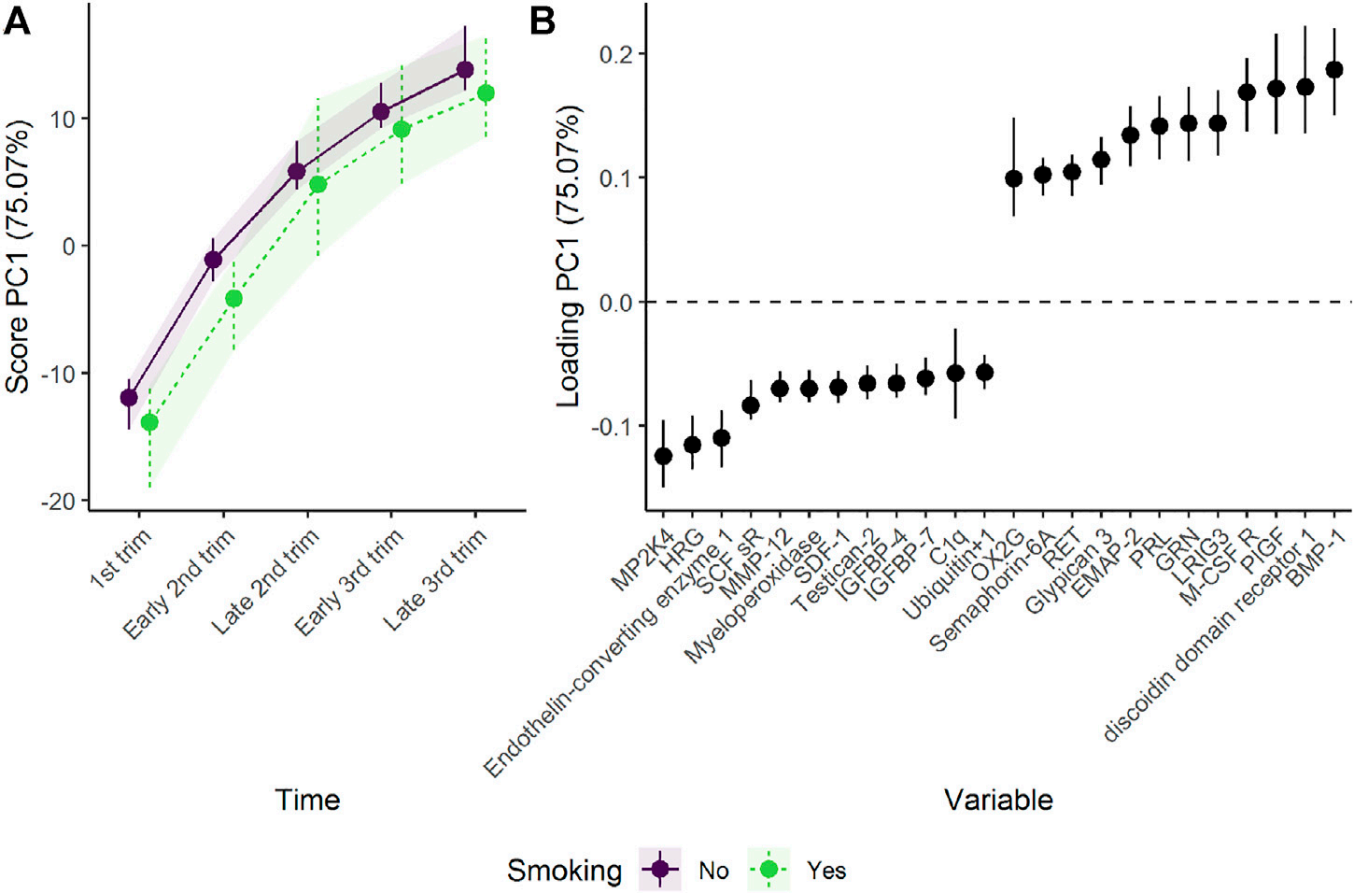
Time development of the plasma proteome throughout pregnancy in smoking and non-smoking women as **(A)** scores and **(B)** loadings. The plasma level of proteins with high loading is increasing when the scores increase and *vice versa*. Only the 12 proteins with highest and lowest loadings, separated by the vertical dotted line, are shown due to the large number of assessed proteins.

Analysis of the combined effect of time and group shows that smoking and non-smoking women demonstrate similar development in plasma proteome in pregnancy, with a tendency to lower scores for the smoking group (Figure 4). The parallel lines in Figure 4 suggest that the differences between the groups are stable over time, with somewhat lower levels of proteins such as BMP-1 and higher levels of proteins such as MP2K4 in smoking women. However, the confidence intervals are overlapping, suggesting that the effect of time is stronger than the effect of smoking, and no group specific development is evident.

**FIGURE 5:**
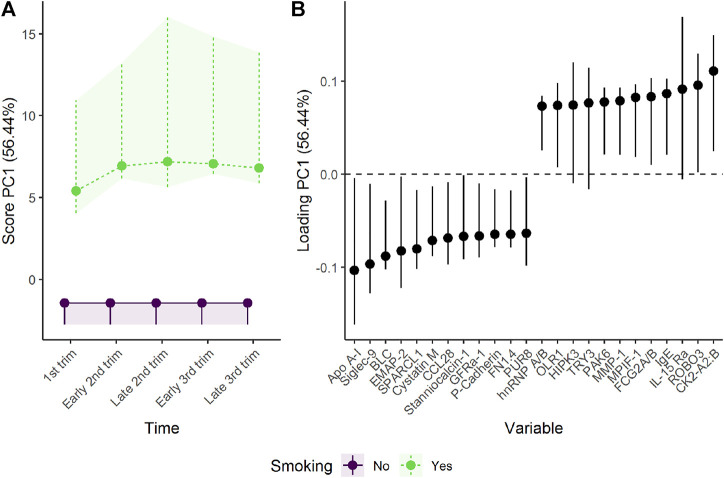
Time development of the plasma proteome throughout pregnancy in smoking and non-smoking women as **(A)** scores and **(B)** loadings. The time development of the non-smoking women has been removed to highlight the effect of smoking. The plasma level of proteins with high loading is increasing when the scores increase and *vice versa*. Only the 12 proteins with highest and lowest loadings, separated by the vertical dotted line, are shown due to the large number of assessed proteins.

Separating the effect of time and group changes the focus from common trajectories to divergent trajectories. The isolated time development of the non-smoking group, acting as reference, is similar to the time development of the combined group shown in Figure 2. The isolated group and time-group effect demonstrates how the plasma proteome of smoking women diverge from non-smoking women during pregnancy (Figure 5). The first component shows a stable and reliable difference between the two groups, with higher scores for the smoking women. Higher scores corresponds to higher plasma levels of proteins with positive loadings and *vice versa*. Thus, smoking women showed higher levels of proteins such as casein kinase II 2-alpha’:2-beta heterotetamer (CK2-A2:B) and roundabout homolog 3 (ROBO3), and lower levels of proteins such as apolipoprotein A-I (Apo A-I) and siglec-9. Apolipoprotein A-I is an important constituent of high-density cholesterol, and is known to be decreased by smoking (Richard et al., 1997; Meenakshisundaram et al., 2010; Slagter et al., 2013).

The ability to adjust for covariates is one of the main advantages of (RM-)ASCA^+^ when compared to other multivariate methods such as PLS. Continuous covariate adjustment was first introduced with ASCA^+^ and with RM-ASCA^+^ this ability has been extended to longitudinal data. For longitudinal trials, adjusting for covariates can offer both more precise and less biased effect estimates, and increase statistical power. Although covariate adjustment can be achieved for methods such as PLS by including it as part of data preprocessing, the ASCA^+^ framework leverages the users’ existing intuitions and knowledge of both linear regression and PCA together in a cohesive approach. With RM-ASCA^+^ the effect of BMI can be isolated by including BMI as a covariate in the regression model, but not in the effect matrices subjected to PCA. The effect of BMI is thus presented as ordinary *β* coefficients (Supplementary Figure S6). The *β* coefficients are the same regardless of whether the time and group effects are assessed separately or not, and represent the adjustment for BMI. High BMI was associated with higher plasma levels of leptin, and the complement components C1s and C5a. In contrast, lower levels of kallistatin, soluble receptor for advanced glycation end products (sRAGE) and neural cell adhesion Molecule (Nr-CAM) were observed with increasing BMI. Obesity is related to low-grade inflammation with lower levels of both the anti-inflammatory kallistatin (Zhu et al., 2013; Frühbeck et al., 2018) and the cardioprotective sRAGE (Norata et al., 2009), and leptin is strongly linked to obesity and correlate with body fat percentage (Obradovic et al., 2021). In addition, the strong effect of BMI on leptin, IGFBP2, and SHBG is in line with previous research on plasma proteomics (Goudswaard et al., 2021).

#### 3.1.3 Does the plasma proteome of pregnancies that are later complicated by early- (EO-) or late-onset (LO-) preeclampsia (PE) follow distinct developmental trajectories?

To assess the developmental trajectories of preeclamptic women, the full data sets of Erez et al. (2017) and Tarca et al. (2019) were used. In total, 572 plasma samples were included for analysis. Of 199 participants, 33 (17%) developed early-onset preeclampsia (EO-PE) and 76 (38%) developed late-onset preeclampsia (LO-PE). For the different time points, 151 (12% EO-PE and 27% LO-PE), 157 (16% EO-PE and 39% LO-PE), 135 (20% EO-PE and 54% LO-PE), and 129 (13% EO-PE and 56% LO-PE) samples were analyzed. The disease groups were coded in the group column with the controls acting as reference and the previous regression formula was similar to the previous example (Section 3.1.1) except that the BMI term was removed. To isolate the potentially distinct trajectories of the preeclamptic pregnancies, the time and group effect matrices were separated by setting separate_effects = TRUE. The RM-ASCA^+^ model was thus initialized as

mod <- ALASCA(

df = df,

formula = value ∼ time*group + (1|ID),

separate_effects = TRUE,

scale_function = “sdt1”,

validate = TRUE

)

The corresponding design matrix is shown in Supplementary Table S3.

**FIGURE 6:**
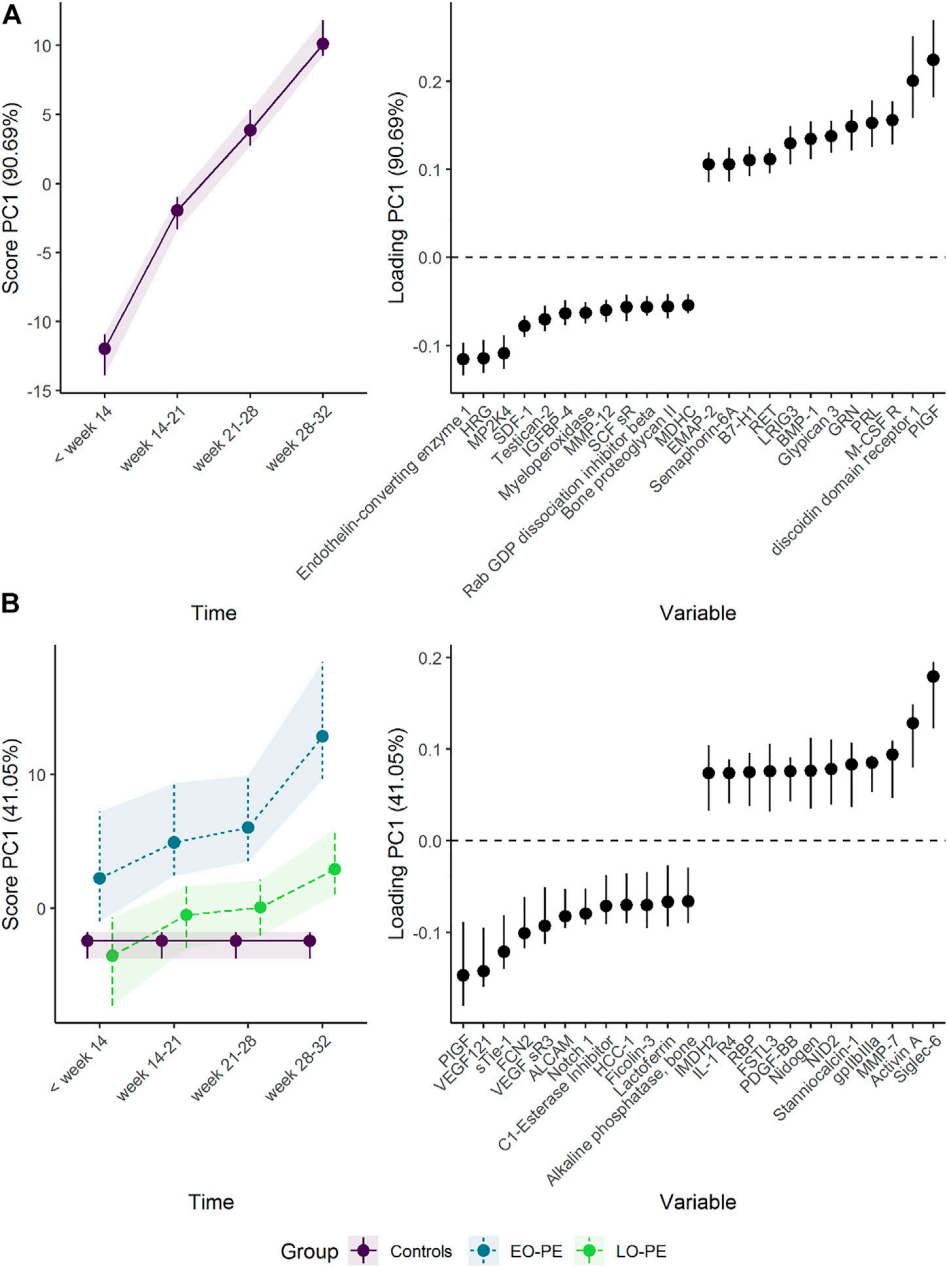
Time development of the plasma proteome throughout pregnancy in **(A)** healthy women and **(B)** women developing early-onset (EO-) or late-onset (LO-) preeclampsia (PE). The time development of healthy women is isolated in the upper panels, whereas the lower panels visualize how the plasma proteome differs between the groups. The plasma level of proteins with high loading is increasing when the scores increase and *vice versa*. Only the 12 proteins with highest and lowest loadings, separated by the vertical dotted line, are shown due to the large number of assessed proteins.

Women developing EO-PE showed lower plasma levels of proteins such as PlGF, VEGF-121, and soluble tyrosine-protein kinase receptor Tie-1 (sTie-1), and higher plasma levels of proteins such as Siglec-6, activin A, and matrilysin/MMP-7 (Figure 6 and Supplementary Figure S7). These findings support the original results by Tarca et al. (2019) (Supplementary Figure S8). The differences from the control group were present from early pregnancy for some proteins, and increased steadily as the pregnancy progressed. The development of the reference group is similar as in sections 3.1.1, 3.1.2 except minor changes of scores and loadings due to redefined time points.

Interestingly, women developing LO-PE showed a similar but delayed shift in plasma proteome (Figure 6). It is, however, necessary to also investigate PC2, as PC1 explained only 41% of the group variation. PC2 demonstrates a clear difference between women developing LO-PE, and the remaining women (Supplementary Figure S9). Women developing LO-PE seem to have higher levels of proteins such as MMP-7, RAN and PPID from early pregnancy, and lower levels of proteins such as HSP70, BMP10, and integrin aVb5 (Supplementary Figure S10). These findings are consistent with the original results by Erez et al. (2017). It is useful to visualize the marginal means from the underlying regression models when a protein has strong loading on multiple PCs and there are robust differences in score in the corresponding PCs. From Supplementary Figures S7, S10, it can be seen that women developing PE had clearly higher MMP-7 throughout pregnancy.

### 3.2 Example 2: Randomized intervention with repeated measurements

#### 3.2.1 How is the metabolomic response in breast cancer affected by adding bevacizumab to standard neoadjuvant chemotherapy?

In contrast to the previous example with observational data, studies with randomized intervention assume that the groups are equal prior to intervention. Thus, the regression model should not include a main effect for treatment (Twisk et al., 2018). A regression model with a time effect, a time-group interaction, and a random intercept can in R be defined as value∼time + time:group + (1|ID). By default, however, the interaction term between time and group (time:group) will include the interaction between the first time point (i.e., baseline) and group, which has to be removed. This can be achieved by providing equal_baseline = TRUE to the ALASCA() function. Thus, the function call

mod <- ALASCA(

df = df,

formula = value ∼ time + time:group + (1|ID),

equal_baseline = TRUE,

scale_function = “sdt1”,

validate = TRUE

)

reproduce the findings of Madssen et al. (2021). The corresponding design matrix is shown in Supplementary Table S4.

To illustrate how scaling and validation strategy impact the analysis, the model was generated for all 16 combinations of scaling (sdall, sdt1, sdref, and sdfref1), resampling (bootstrap and jack-knife), and extraction of effect matrices (combined and separate). The bootstrap and jack-knife samples were reused for each model to make the results comparable.

**FIGURE 7:**
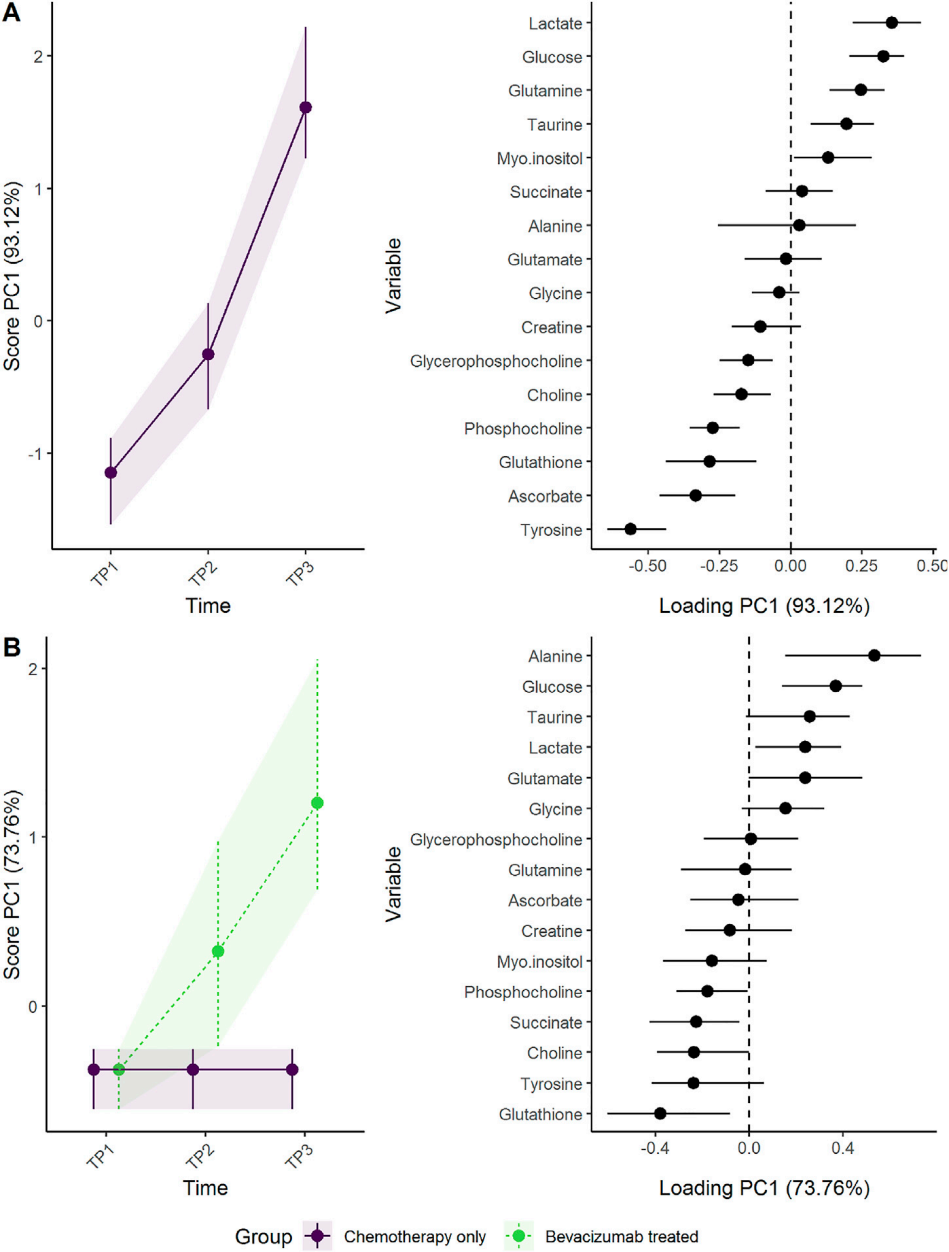
Time development of tumor biopsy metabolome before and during cancer treatment. **(A)** The time development of the participants receiving chemotherapy only is isolated in the upper panels, whereas **(B)** the lower panels visualize how the metabolome differs between the groups. The levels of metabolites with high loading is increasing when the scores increase and *vice versa*.

To assess the effect of adding the drug bevacizumab to standard neoadjuvant chemotherapy to treat breast cancer, the effect matrix for time and the effect matrix for time-group interaction were analyzed separately by PCA (Figure 7). The addition of bevacizumab led to higher concentrations of alanine, glucose, and lactate, and lower concentrations of gluthatione, succinate, and phosphocoline. The increased alanine and glucose levels, and decreased gluthatione levels, were statistically significant at *T*_3_ following bevacizumab treatment in univariate models (Supplementary Figure S11) and the residuals showed acceptable normal distribution (Supplementary Figure S12). These results are discussed in detail by Madssen et al. (2021). ALASCA also allows the results to be displayed as a more classical ASCA analysis, by plotting the first and second PC against each other, as in Supplementary Figure S13.

**FIGURE 8:**
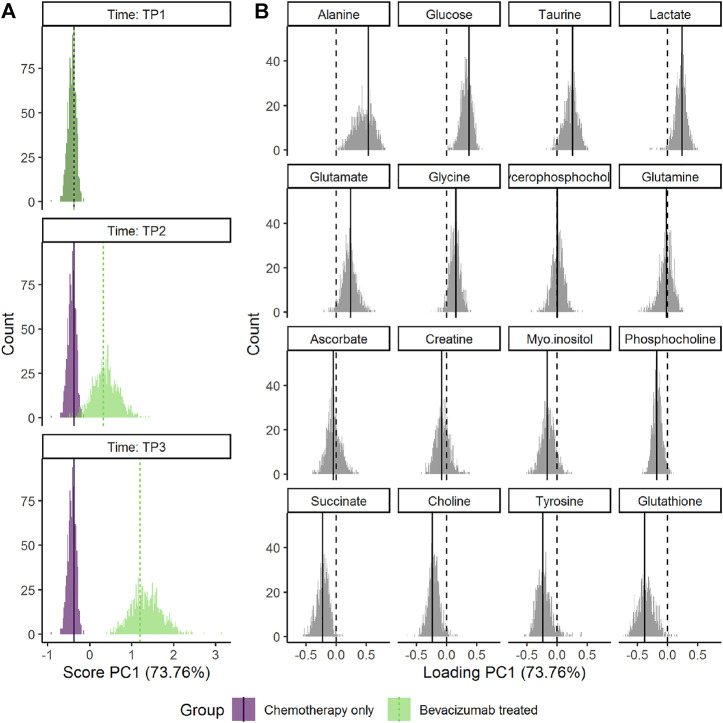
Distribution of the bootstrapped parameters **(A)** scores and **(B)** loadings for the RM-ASCA^+^ model shown in Figure 7. Main model estimates are shown as vertical line. The dotted lines mark zero. The plot was made with the plot(…, type = “histogram”) function.

The choice of scaling and validation strategy has strong impact on uncertainty estimates (Supplementary Figures S14–S16). Jack-knife resulted in markedly smaller CIs for both scores and loadings than bootstrap. The choice of scaling does not alter how the results are interpreted but using baseline samples for scaling (sdt1 or sdreft1) enhanced the separation of the groups at the third time point. ALASCA provides two additional visualizations of the validation results: either the scores and loadings for each individual iteration (Supplementary Figure S17) or the distribution of scores and loadings as histograms (Figure 8).

In general, Timmerman et al. (2015) advice that “scaling factors should be free from the effect of interest.” The argument is that if the effect of interest actually increases between-group variation, then we have to avoid that this effect is damped by scaling. I.e., the between-group variation introduced by experimental manipulation should not be part of the scaling factor. In this specific example with a randomized trial, the baseline measurements constitute a subset of data where no such between-group variation has yet been introduced. In other cases, however, it may be less clear which groups that are affected by the experimental condition of interest. In addition, the scaling factor must be based on a sufficiently large group. In this paper, we are primarily using the baseline measurement for scaling to balance the need for a sample free from the effect of interest (typically the effect of time and time-group interaction) and sample size. In example 3, however, where a healthy and a diseased population are compared at a single time point and where the disease is manifest, the scaling factor is based on the healthy controls only.

Bootstrapping seems the preferable resampling strategy despite jack-knifing resulting in smaller CIs and clearer separation between groups. Targeted studies are needed to assess the performance and coverage of specific validation strategies for (RM-)ASCA^+^, and the most conservative approach seems reasonable until such studies emerge. A possible explanation for the smaller CIs from jack-knife may be that bootstrapping “‘shakes’ the original data more violently than jackknifing” (Efron and Hastie, 2016, p. 161); on average, bootstrapping leaves out approximately 37% of the participants compared to 14% for jack-knife when 1/7 participants are excluded. Many refined strategies exist for resampling and CI calculation for multilevel models and may be implemented in later versions of ALASCA when the strengths and weaknesses have been thoroughly mapped (van der Leeden et al., 2008). Similarly, permutation tests exist in exact or approximate form for general ASCA models and provide means to calculate *p* values for model terms and interactions (Anderson and Braak, 2003; Bertinetto et al., 2020), and may be implemented in ALASCA when their performance under various model design have been thoroughly explored.

#### 3.2.2 How does the metabolomic response in breast cancer differ between responders and non-responders receiving neoadjuvant chemotherapy with or without bevacizumab?

To investigate whether the metabolomic changes in tumors from patients having a good response to either chemotherapy alone or chemotherapy+bevacizumab differed from non-responders, a main effect for response and a three-way interaction between time, group, and response was added. In R, the model can be specified as value ∼ time + response + time:response + time:group + time:group:response + (1|ID). Since equal_baseline = TRUE, the *treatment* groups are similar at baseline, whereas the *response* groups can differ. In this case, the effect matrix is specified manually. If not, the response effect would be separated as for BMI in example 1. The ALASCA() call was:

mod <- ALASCA(

df = df,

formula = value ∼ time + response +

time:response + time:group +

time:group:response + (1|ID),

equal_baseline = TRUE,

effects = “time + response + time:response +

time:group + time:group:response”,

scale_function = “sdt1”,

validate = TRUE

)

The corresponding design matrix is shown in Supplementary Table S5.

**FIGURE 9:**
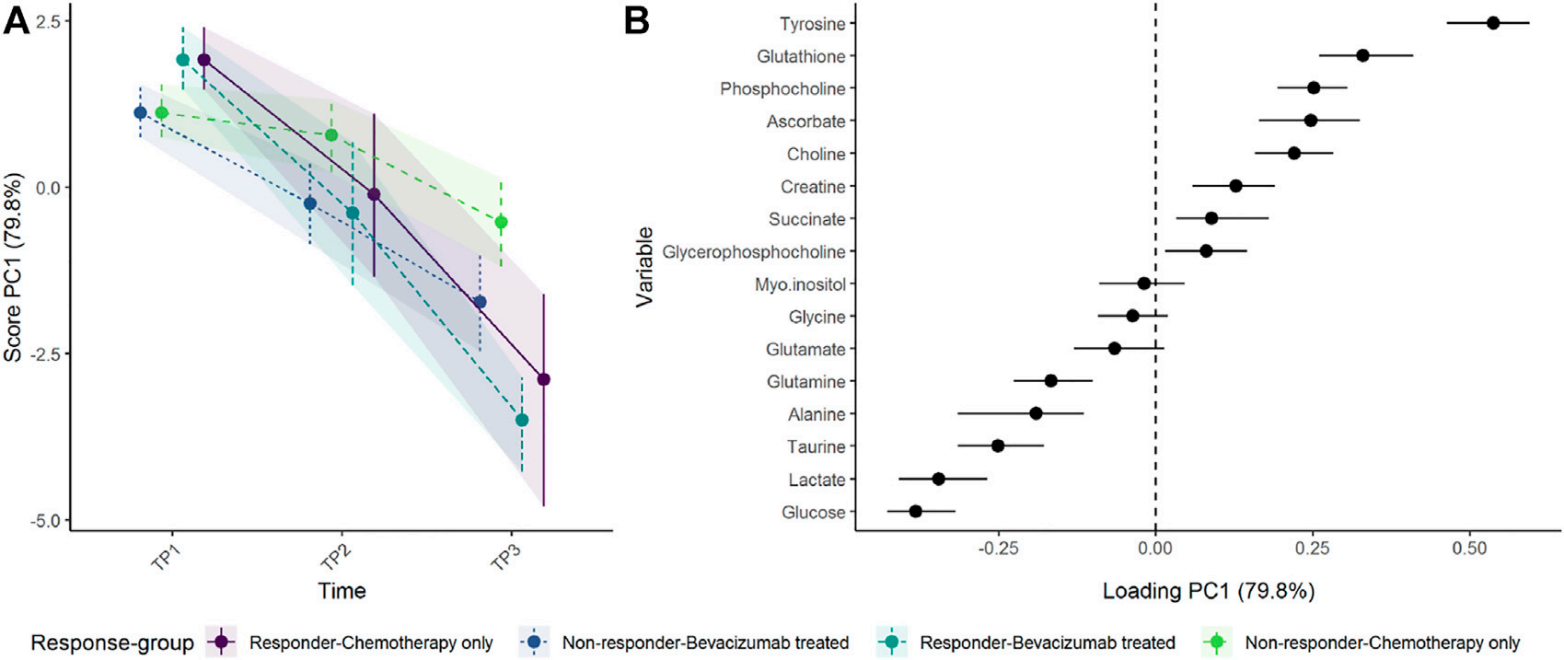
Time development of tumor biopsy metabolome before and during cancer treatment, in responders and non-responders as **(A)** scores and **(B)** loadings. Since participants were randomized to treatment but not response, the baseline levels are equal for all participants within each response groups. Non-responders receiving chemotherapy only show the smallest change of metabolome. The level of metabolites with high loading is increasing when the scores increase and *vice versa*.

The regression model including a three-way-interaction between time, response, and treatment showed that responders had somewhat higher concentrations of tyrosine and glutathione, and lower concentrations of glucose and lactate at baseline and showed a larger shift in metabolomic profile than non-responders (Figure 9). After 12 weeks of treatment (*T*_2_), the metabolomic shift seems similar in the reponder group as well as non-responders receiving bevacizumab. At 24 weeks, however, the responders had the largest change in metabolic profile, followed by non-responders receiving bevacizumab, whereas non-responders receiving chemotherapy only had the smallest change.

One should note that the baseline levels shown in Figure 9 reflect a more complex statistical model than the previous example, where the treatment groups shared the same baseline. Since the tumors from responders and non-responders may have had some distinct properties from the beginning, the baseline levels of responders and non-responders are allowed to vary, whereas the baseline levels of the treatment groups are kept equal. Thus, the three-way interaction between time, treatment, and response could not have been reproduced by simply creating four groups (treatment×response) and using the same regression model as above (value∼time + time:group + (1|ID)).

### 3.3 Example 3: Megavariate data

#### 3.3.1 Does skin gene expression differ between patients with systemic sclerosis (SSc) and healthy controls?

Since control samples were only available for a single time point, skin gene expression in healthy controls were compared to patients with limited or diffuse SSc at baseline. Reduction of dimensions by PCA was applied due to the size of the data set.

Although the ALASCA package is primarily designed for longitudinal data sets, it also supports ordinary linear models without random effects. When there is no time term in the regression formula, the first term will be used as abscissa. Gender and age were included as covariates to demonstrate adjustment of categorical and continuous variables. In R, the regression model can be defined as value∼disease + gender + age:

mod <- ALASCA(

df = df,

formula = value ∼ disease + gender + age,

scale_function = “sdref”,

reduce_dimensions = TRUE,

validate = TRUE

)

The corresponding design matrix is shown in Supplementary Table S6.

**FIGURE 10:**
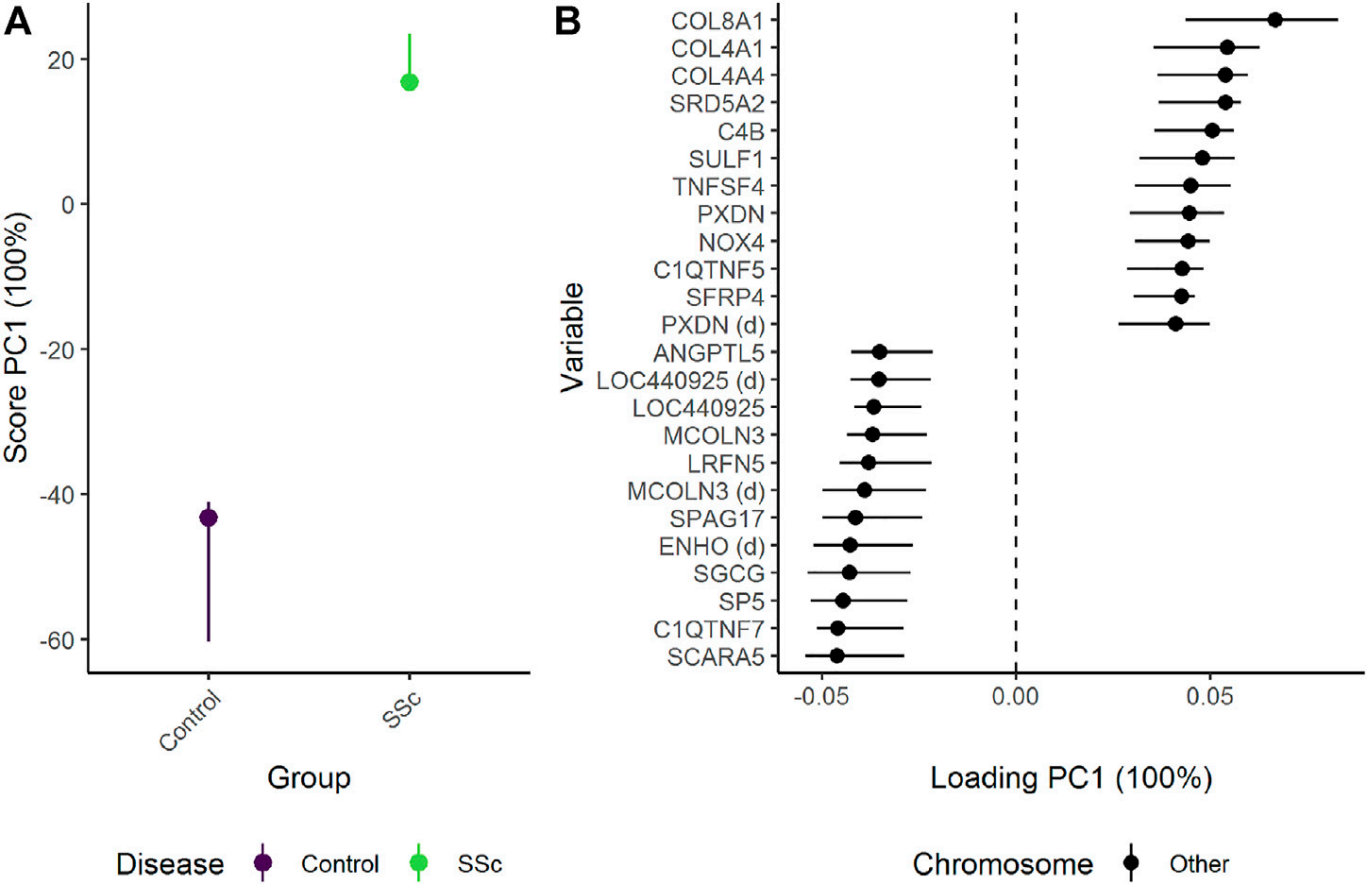
The difference in skin biopsy transciptome between healthy controls and patients with systemic sclerosis (SSc) as **(A)** scores and **(B)** loadings. The effects of age and gender have been adjusted for (Figure 11). The expression of genes with high loading is increasing when the scores increase and *vice versa*. Only the 12 genes with highest and lowest loadings, separated by the vertical dotted line, are shown due to the large number of assessed genes.

ALASCA can be used to compare multivariate data from experimental designs with single measurements and adjust for confounders such as gender. When only two groups are compared, the difference between the groups is fully explained by PC1 (Figure 10). Patients with SSc showed stronger expression of several genes related to collagen alpha proteins such as COL8A1, COL4A1, and COL4A4. In contrast, the healthy controls showed stronger expression of genes such as SCARA5 (Scavenger Receptor Class A Member 5), C1QTNF7 (Complement C1q Tumor Necrosis Factor-Related Protein 7), SP5 (Transcription Factor Sp5), SGCG (sarcoglycan gamma), and ENHO (Energy Homeostasis-Associated Protein). The genes with highest and lowest loading showed some overlap with the genes with the highest/lowest fold-change as reported in the original study, but ALASCA also identified several new genes of possible interest (Supplementary Figure S18). In addition, the original study did not adjust for gender and age.

**FIGURE 11:**
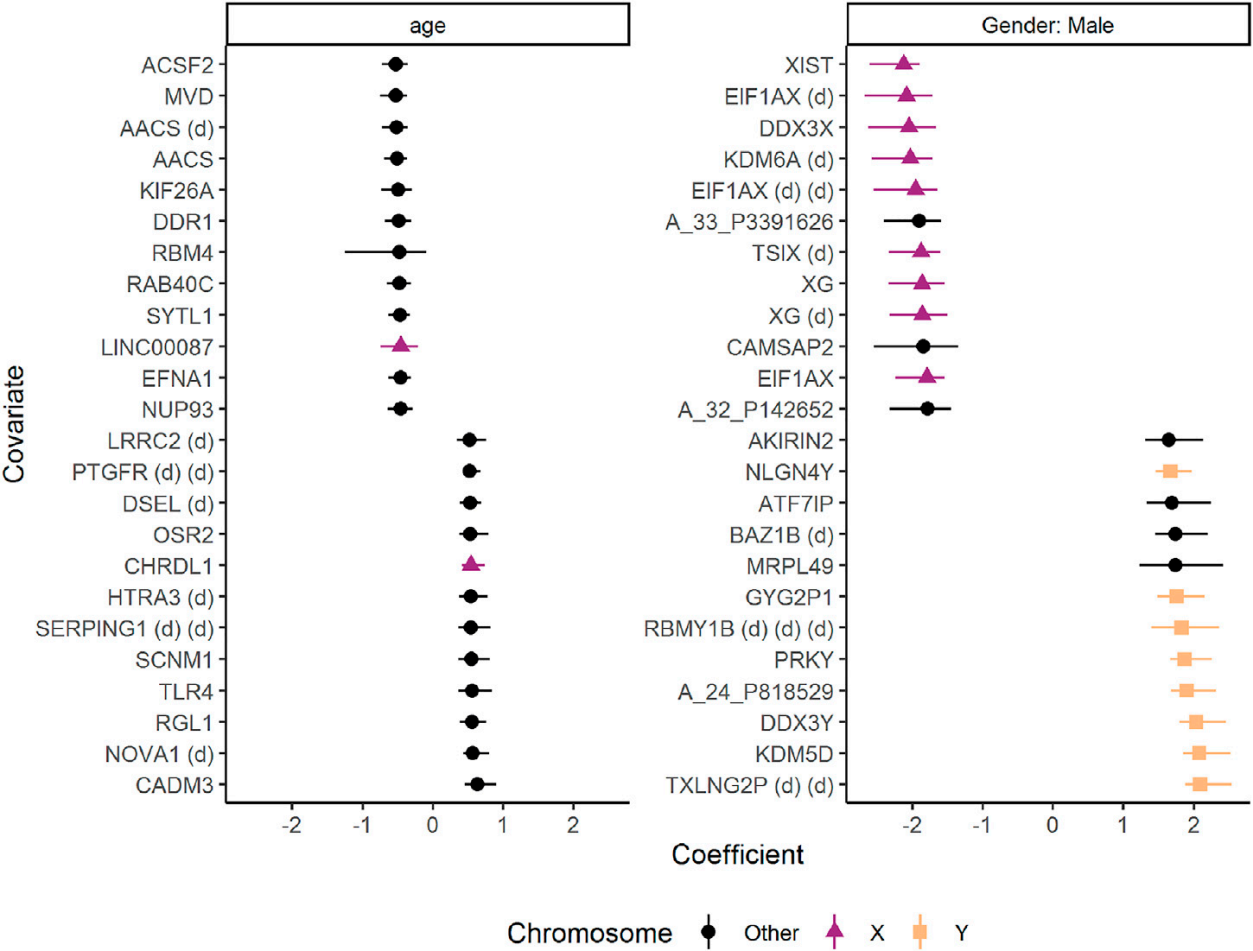
The effects of age and gender on gene expression in skin biopsies from healthy controls and patients with systemic sclerosis. The coefficients are regression coefficients from linear regression models, colored by chromosome location. Some genes were associated with mulitple probes, and are marked with “(d)” to avoid duplicated names. The error bars reflect 95% confidence intervals from bootstrapping. Only the 12 genes with highest and lowest coefficients are shown. The figure was made with the plot(…, type = “covars”) function.

Many of the genes differently expressed in males and females were located on the sex chromosomes (Figure 11). Male participants had stronger expression of genes such as TXLNG2P (Taxilin Gamma Pseudogene, Y-Linked), Lysine Demethylase 5D (KDM5D), and DDX3Y (DEAD-Box Helicase 3, Y-Linked). Females, on the other hand, showed stronger expression of genes such as XIST (X Inactive Specific Transcript), EIF1AX (Eukaryotic Translation Initiation Factor 1A, X-Linked), and DDX3X (DEAD-Box Helicase 3, X-Linked). Increasing age was associated with stronger expression of genes such as CADM3 (Cell Adhesion Molecule 3) and NOVA1 (NOVA Alternative Splicing Regulator 1), whereas genes such as ACSF2 (Acyl-CoA Synthetase Family Member 2) and MVD (Mevalonate Diphosphate Decarboxylase) showed the opposite pattern.

The default settings in the ALASCA package are suggestions and should not be treated as authoritative recommendations. The user’s choice of parameters and settings should be informed by the research question and the data. For example, by reducing the number of variables through PCA as in this example, one improves efficiency at the cost of accuracy. Currently, there are many opinions on how to select the number of necessary components (Abdi and Williams, 2010), and the performance of various methods depends on the nature of the data being studied (Peres-Neto et al., 2005). The number of components selected by the ALASCA package during dimension reduction depends on how much variance wish to retain (by default, reduce_dimensions.limit = 0.95 so that 95% of the variance will be kept). A good strategy would be to compare the results from multiple models with various limits to see how sensitive the results are to that specific parameter. A similar strategy can be employed to gain confidence in other parameters as well.

#### 3.3.2 Does longitudinal skin gene expression differ between patients with limited and diffuse SSc?

The longitudinal skin gene expression from patients with limited or diffuse SSc was assessed with the limited variant as reference group. To reduce the number of variables subjected to regression by applying an initial PCA prior to regression, reduce_dimensions was set to TRUE. As the default PCA algorithm in R sometimes stops due to internal errors, an alternative PCA function can be provided by specifying pca_function (Baglama et al., 2021). The regression model is similar to the final model in Example 1 with separated effects for time and group:

mod <- ALASCA(

df = df,

value ∼ time * group + (1|ID),

scale_function = “sdt1”,

pca_function = “irlba”,

reduce_dimensions = TRUE,

separate_effects = TRUE,

validate = TRUE

)

The corresponding design matrix is shown in Supplementary Table S7.

**FIGURE 12:**
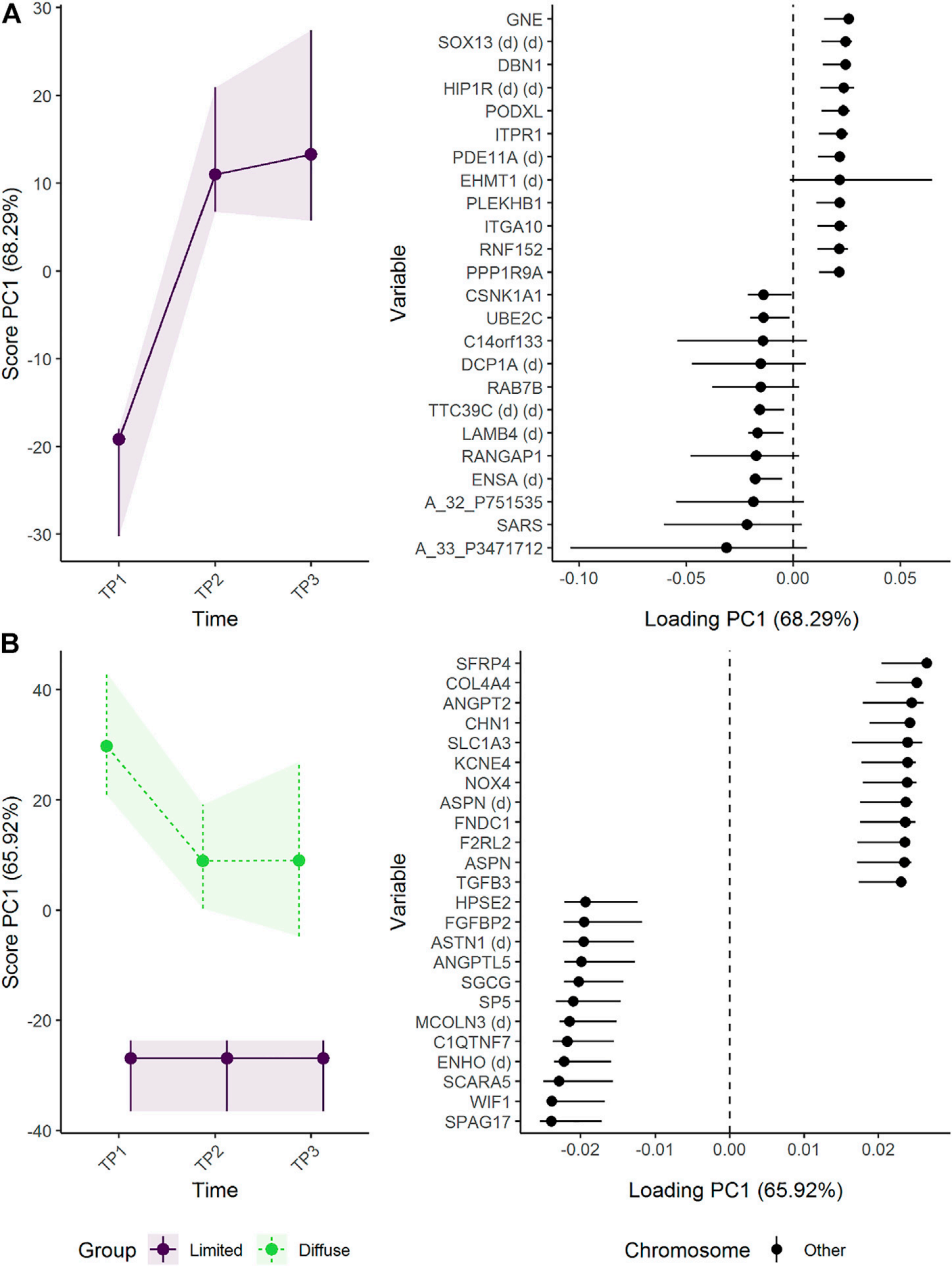
Time development of skin biopsy genome in patients with limited or diffuse systemic sclerosis. **(A)** The time development of patients with limited systemic sclerosis is isolated in the upper panels, whereas **(B)** the lower panels visualize how the skin biopsy genome develop distinctly between the groups. The expression of genes with high loadings is increasing when the scores increase and *vice versa*. Only the 12 genes with highest and lowest loadings, separated by the vertical dotted line, are shown due to the large number of assessed genes.

The initial skin biopsy from patients with limited SSc differed from the two subsequent biopsies with a tendency to increased expression of genes such as GNE (Bifunctional UDP-N-acetylglucosamine 2-epimerase/N-acetylmannosamine kinase), SOX13 (SRY-Box Transcription Factor 13), and DBN1 (drebin 1) with time (Figure 12A). The difference in gene expression between the patient groups was stable over time (Figure 12B). Patients with diffuse SSc showed stronger expression of genes such as SFRP4 (Secreted Frizzled Related Protein 4), ANGPT2 (Angiopoietin 2), and COL4A4 (Collagen Type IV Alpha 4 Chain) than patients with limited SSc. In contrast, genes such as SPAG17 (Sperm Associated Antigen 17), SCARA5, and WIF1 (WNT Inhibitory Factor 1) were more strongly expressed in skin from patients with limited SSc than patients with diffuse SSc. Although SFRP4 was reported to have the highest fold-change between diffuse and limited SSc in the original publication (Skaug et al., 2021), ALASCA identifies several the genes of possible interest (Supplementary Figure S19).

## 4 Conclusion

The (RM-)ASCA^+^ framework offers a flexible and robust method to quickly discover patterns in multivariate data. Advantages with (RM-)ASCA^+^ compared to other methods such as PLS-DA include the possibility to model longitudinal changes from multiple timepoints, to incorporate advanced experimental designs, and to include confounders in the analysis. The ALASCA package for R makes the (RM-)ASCA^+^ available for general use by offering a simple interface to model complex relationships, to scale the data, to perform model validation, and to produce a variety of publication-ready visualizations.

## Data Availability

The original contributions presented in the study are included in the article/Supplementary Material and at https://doi.org/10.6084/m9.figshare.21362979.v1. Further inquiries can be directed to the corresponding author.
