# Supplementary material for: ALASCA: An R package for longitudinal and cross-sectional analysis of multivariate data by ASCA-based methods
Source: Front Mol Biosci. 2022 Oct 26;9:962431. doi: 10.3389/fmolb.2022.962431 (PMC9645785; doi:10.3389/fmolb.2022.962431)
Supplement: Supplementary file 1 [file DataSheet1.pdf]

Supplementary Material

1 SUPPLEMENTARY FIGURES

(**A**) Long format.

ID	time	group	variable	value
1	T1	placebo	CRP	11.2
1	T2	placebo	CRP	15.3
1	T3	placebo	CRP	19.6
1	T1	placebo	Glucose	4.8
1	T2	placebo	Glucose	5.1
1	T3	placebo	Glucose	4.7
2	T1	treatment	CRP	13.6
2	T2	treatment	CRP	9.4
⋮	⋮	⋮	⋮	⋮

(**B**) Wide format.

ID	time	group	CRP	Glucose	...
1	T1	placebo	11.2	4.8	...
1	T2	placebo	15.3	5.1	...
1	T3	placebo	19.6	4.7	...
2	T1	treatment	13.6	11.1	...
2	T2	treatment	9.4	12.0	...
⋮	⋮	⋮	⋮	⋮	⋮

Figure S1. Example of a tables in (**A**) long and (**B**) wide format. In long format, each measurement has a separate row. In wide format, each sample has a separate row, with measurements as columns.

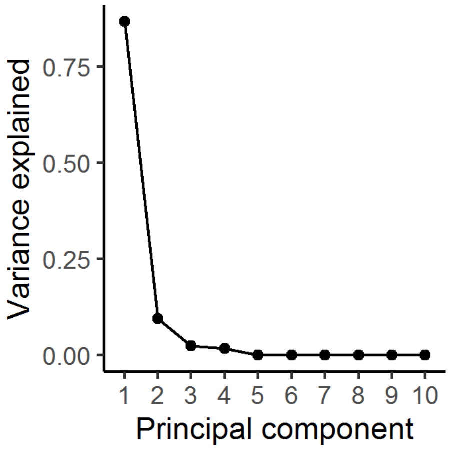

Figure S2. A scree plot showing the proportion of variance explained by each principal component. Scree plots can be made with `plot(..., type = "scree")`.

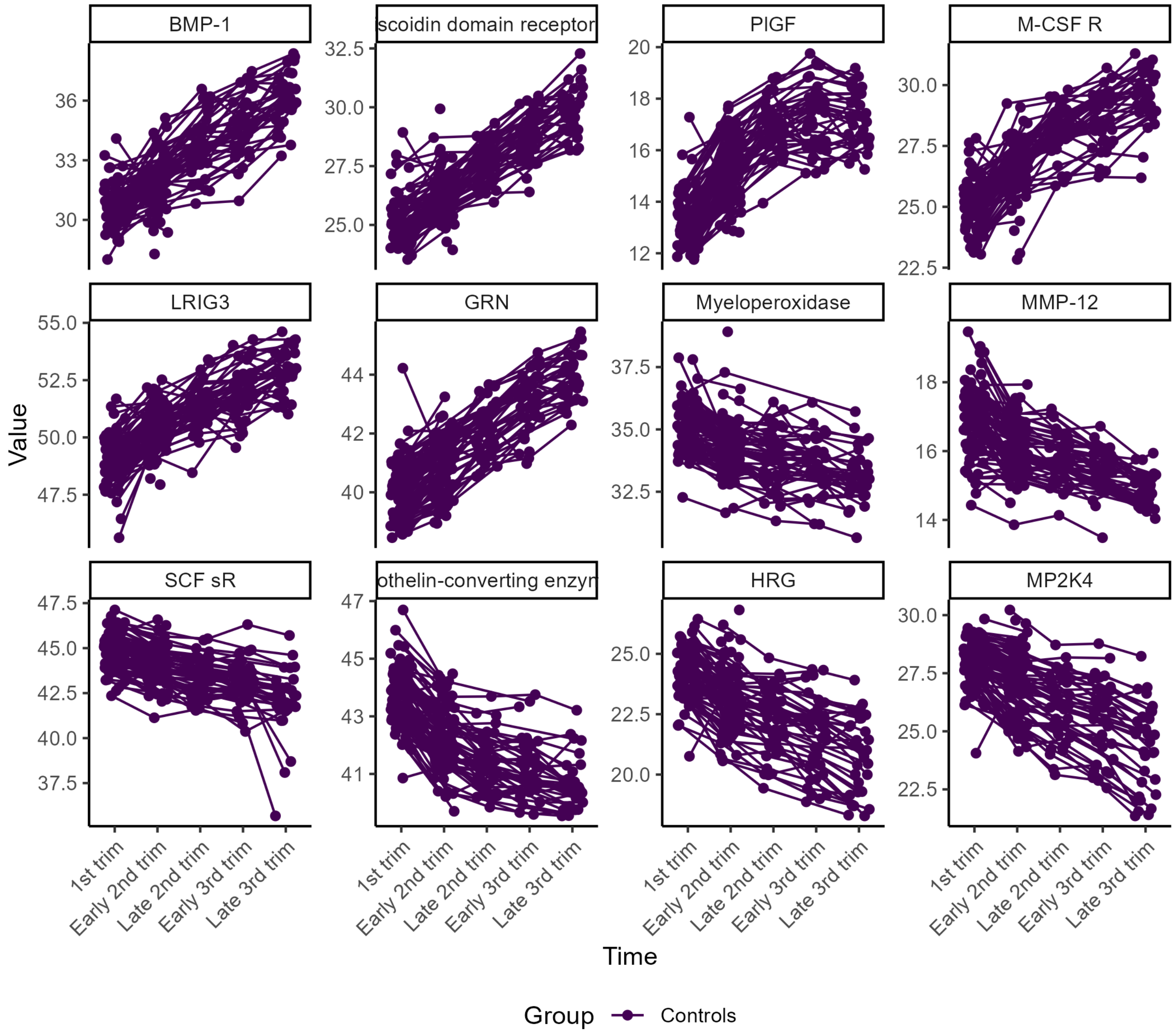

Figure S3. Plasma concentration of selected proteins in log relative fluorescence units, plotted by participant. The plasma concentrations are reflected by the 1st principal component (PC1) shown in Figure 2. Measurements from the same individual are connected by a line. The figure was made with `plot(..., type = "participants")`.

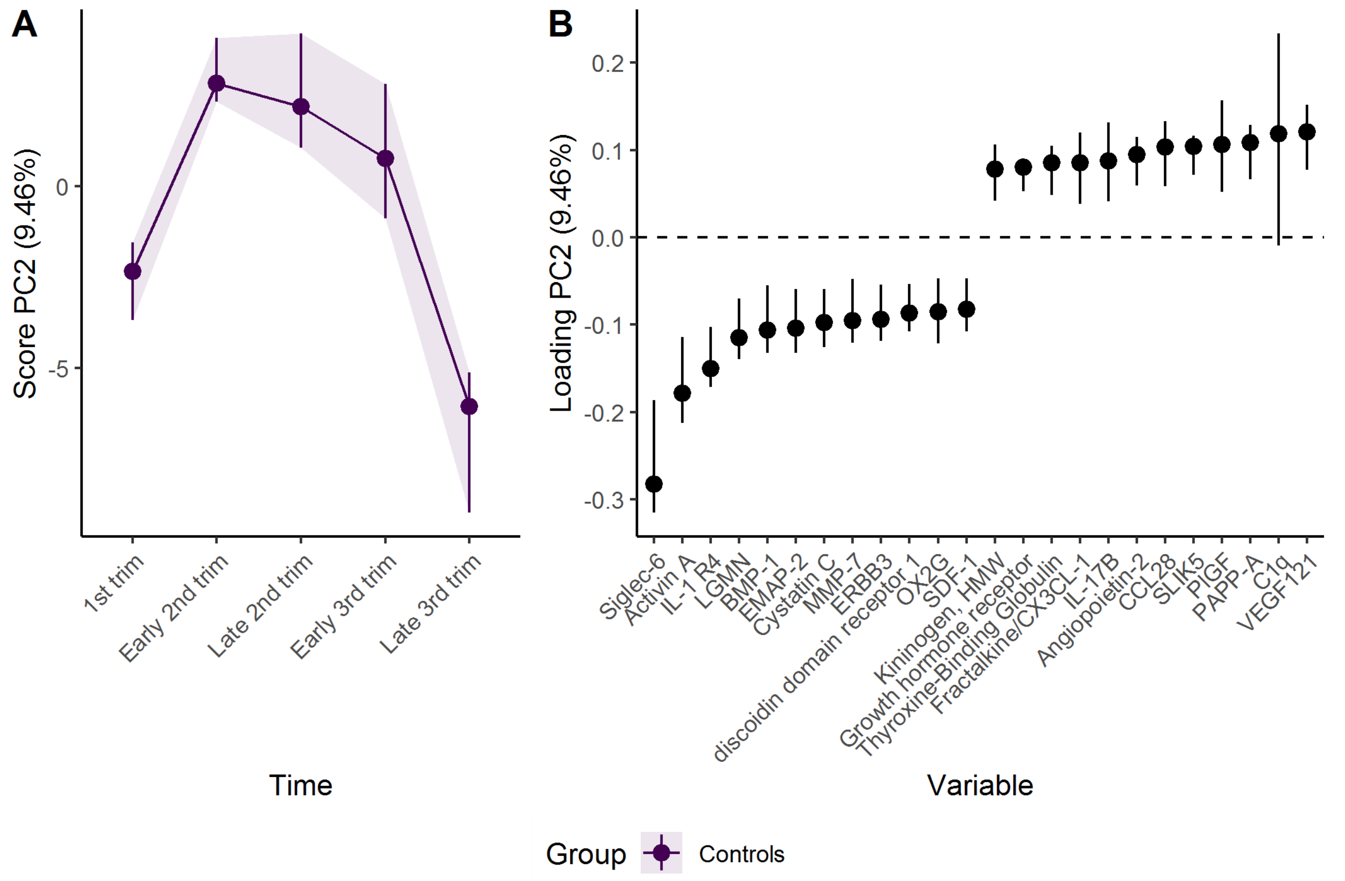

Figure S4. Time development of the plasma proteome throughout pregnancy as (**A**) scores and (**B**) loadings. The plasma level of proteins with high loading is increasing when the scores increase and *vice versa*. Only the 12 proteins with highest and lowest loadings are shown due to the large number of assessed proteins.

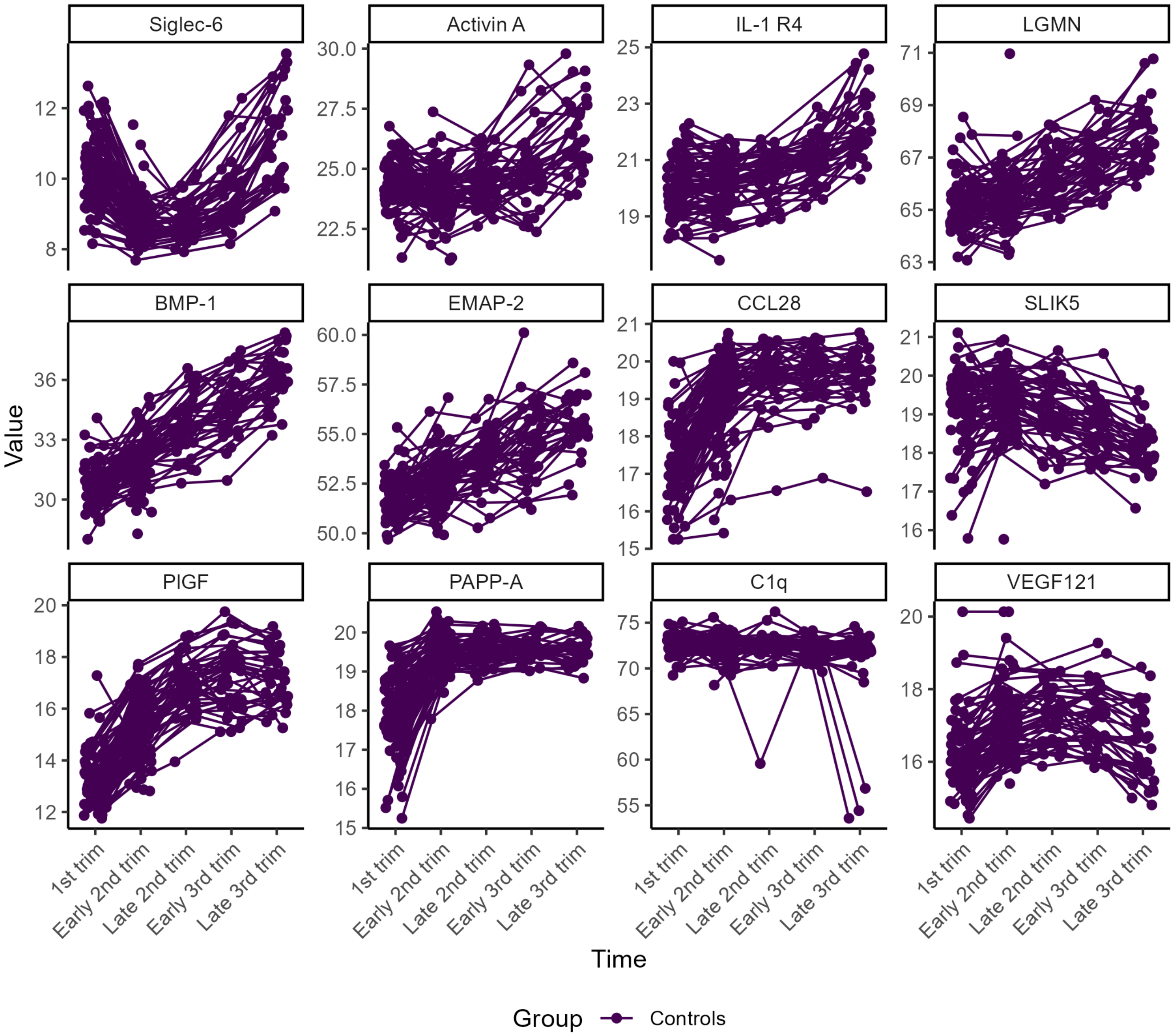

Figure S5. Plasma concentration of selected proteins in log relative fluorescence units, plotted by participant. The plasma concentrations are reflected by the 2nd principal component (PC2) shown in Figure S4. Measurements from the same individual are connected by a line.The figure was made with `plot(..., type = "participants")`.

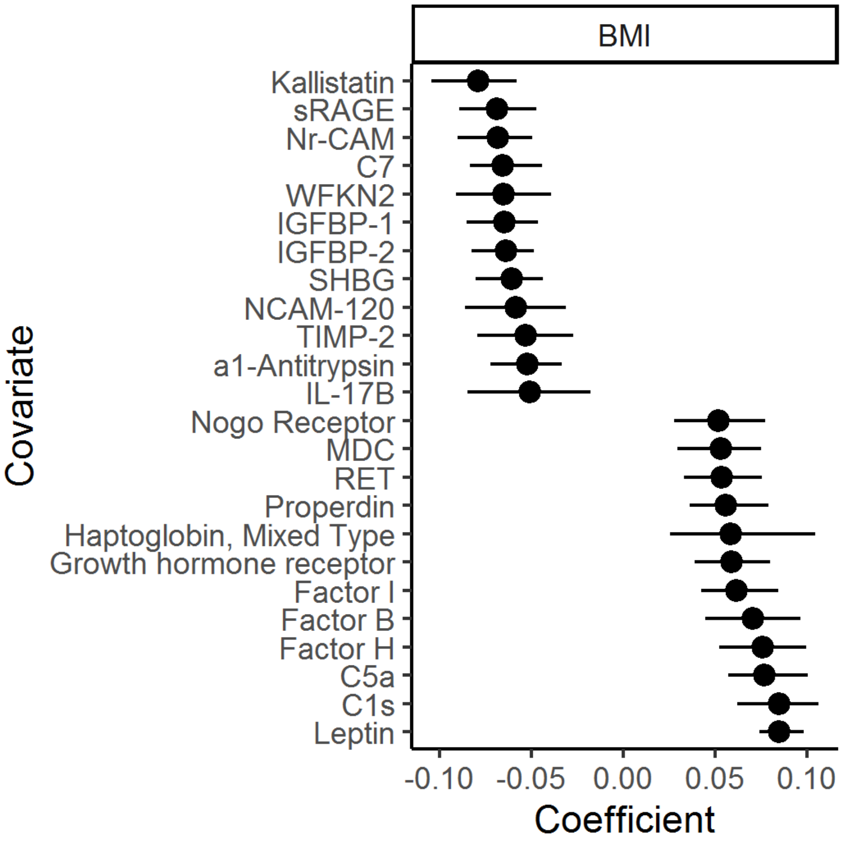

Figure S6. The effects of body mass index (BMI) on protein levels throughout pregnancy. The coefficients are regression coefficients from linear mixed regression models and the error bars reflect 95% confidence intervals from bootstrapping. Only the 12 proteins with highest and lowest coefficients are shown. The figure was made with `plot(..., type = "covars")`.

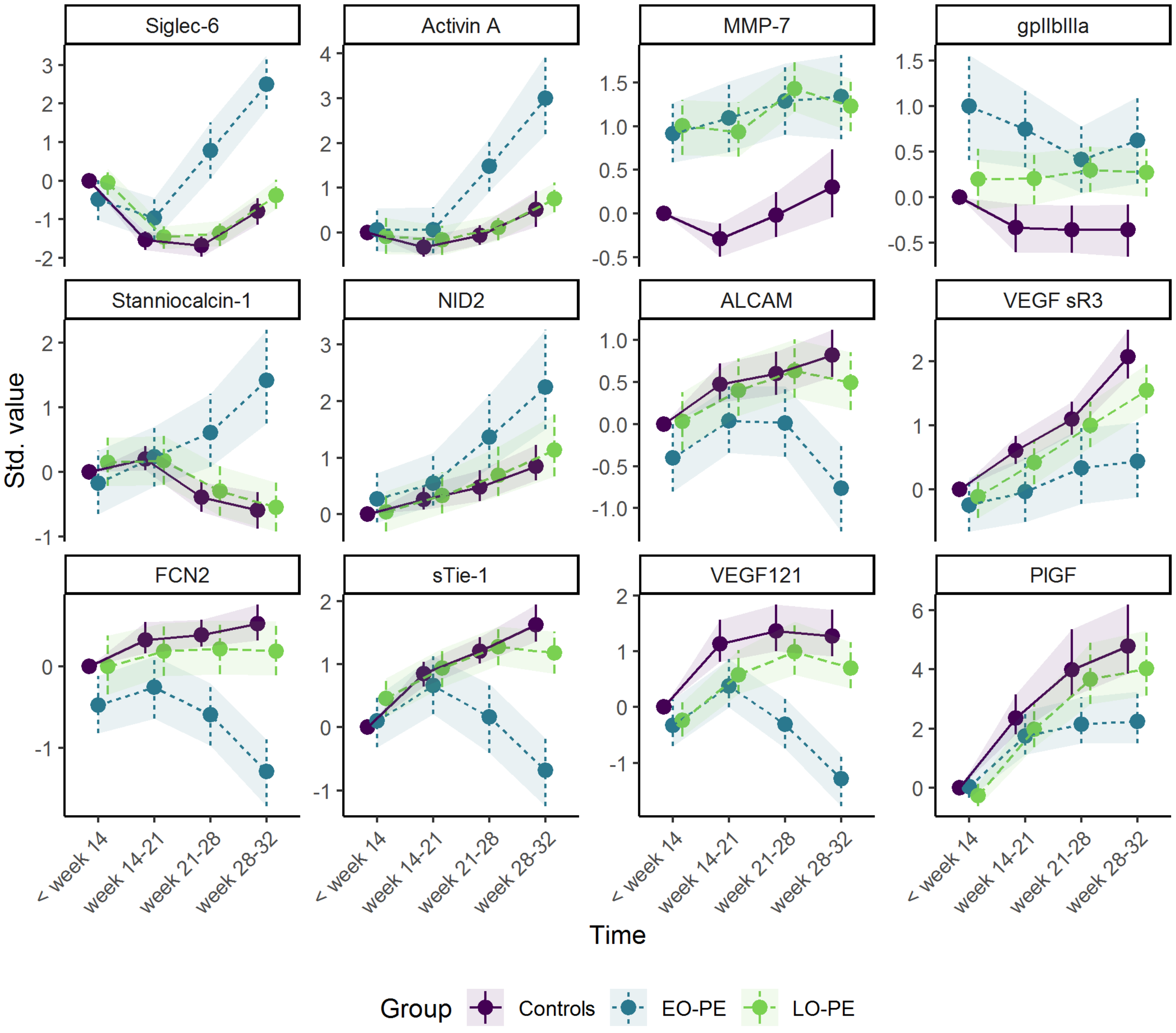

Figure S7. Marginal means for scaled protein concentration for healthy pregnant women (controls) and women developing early-onset (EO-) and late onset (LO-) preeclampsia (PE), calculated from linear mixed models. The intercept has been removed to highlight the robustness of development over time. The plot was made with `plot(..., type = "prediction")`.

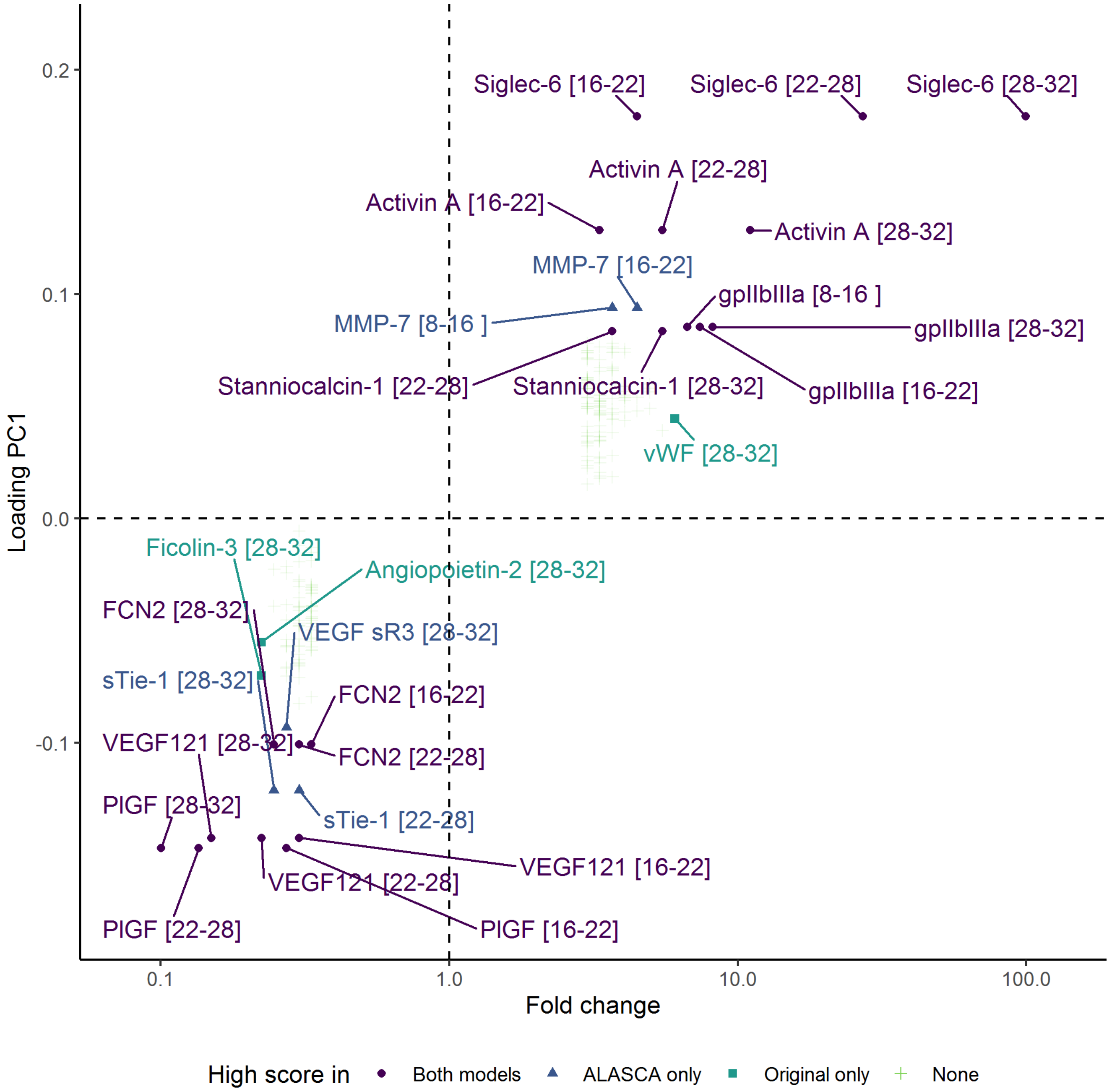

Figure S8. Comparison of RM-ASCA$^+$loadings to fold-change from the original publication. The fold changes are for women with early onset preeclampsia compared to healthy controls at various weeks (in brackets). RM-ASCA$^+$loadings show overall difference between the groups so that only one loading is calculated for each protein. The five proteins with highest and lowest score from each model are labeled.

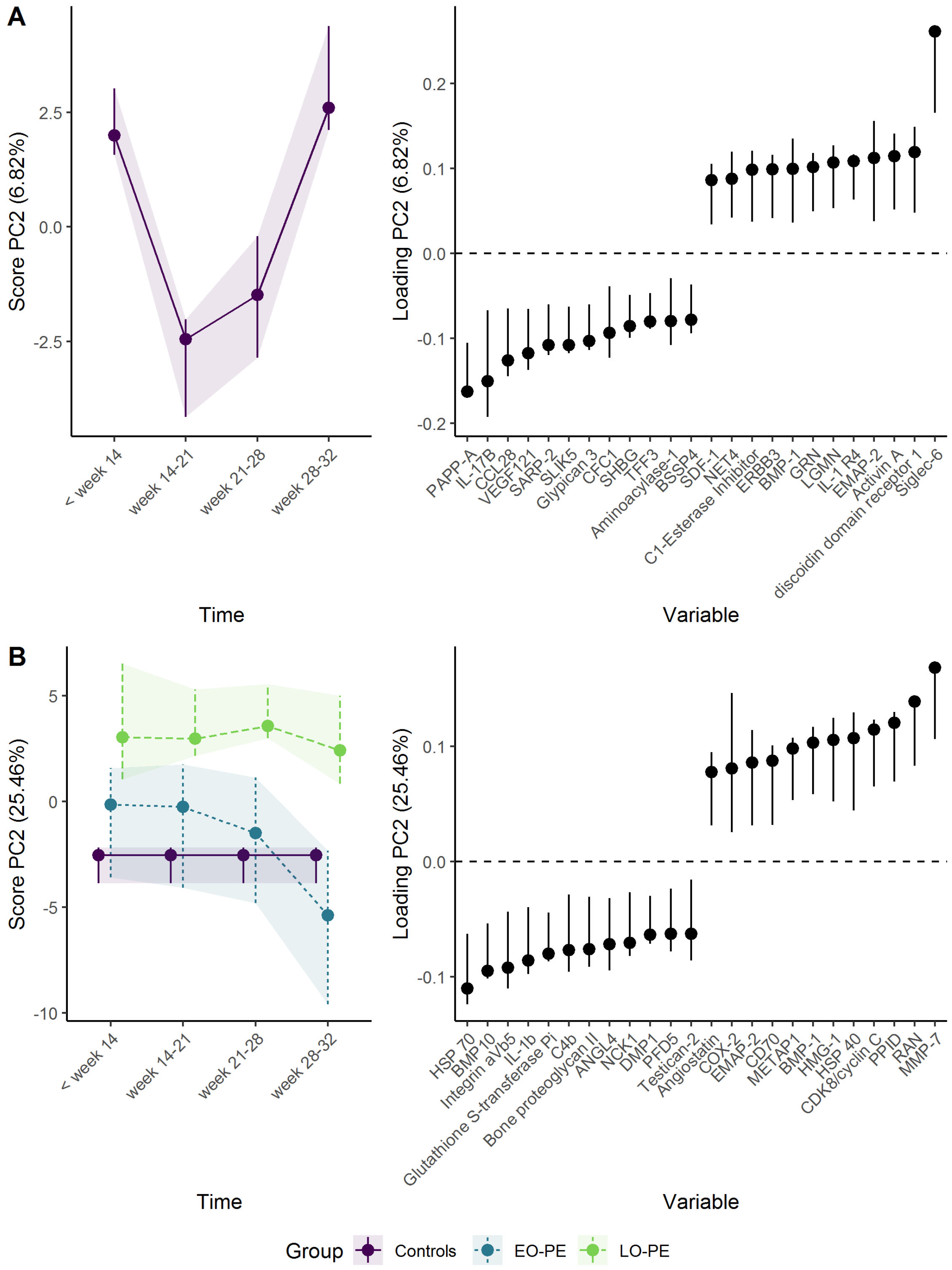

Figure S9. Time development of the plasma proteome in (**A**) healthy pregnant women (controls) and (**B**) women developing early-onset (EO-) or late-onset (LO-) preeclampsia (PE). The plasma level of proteins with high loading is increasing when the scores increase and *vice versa*. The time development of healthy women has been isolated (**A**) to highlight the distinct proteome changes in women developing PE (**B**). Only the 12 proteins with highest and lowest loadings are shown due to the large number of assessed proteins.

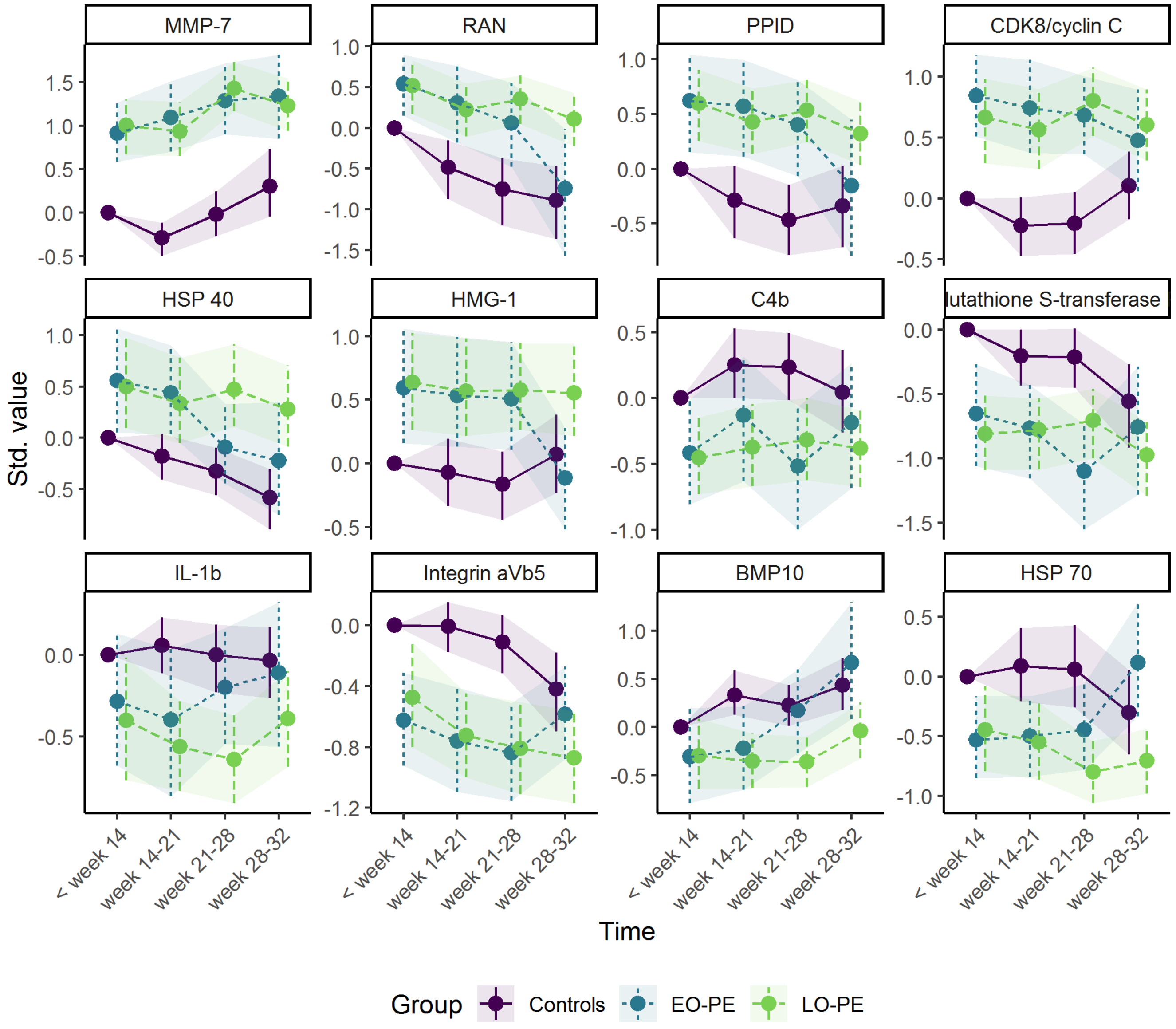

Figure S10. Marginal means for scaled protein concentration for healthy pregnant women (controls) and women developing early-onset (EO-) and late onset (LO-) preeclampsia (PE), calculated from linear mixed models. The intercept has been removed to highlight the robustness of development over time. The plot was made with `plot(..., type = "prediction")`.

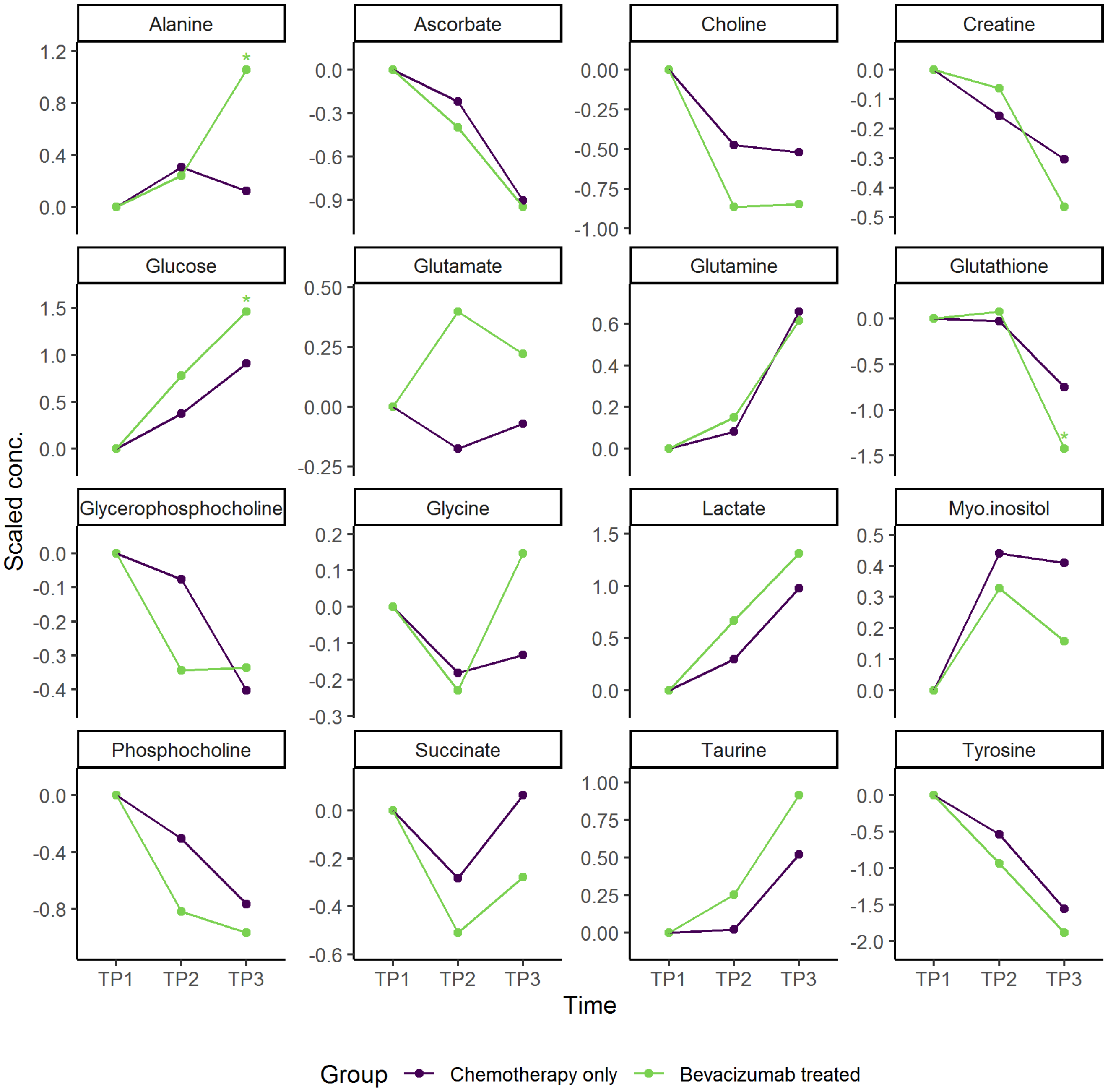

Figure S11. Marginal means for tumor biopsy metabolite levels estimated from linear mixed models. The concentrations have been normalized, and the p-values are calculated for change from baseline with the Satterthwaite's degrees of freedom method and have been corrected for multiple comparisons with the Benjamini-Hochberg procedure.

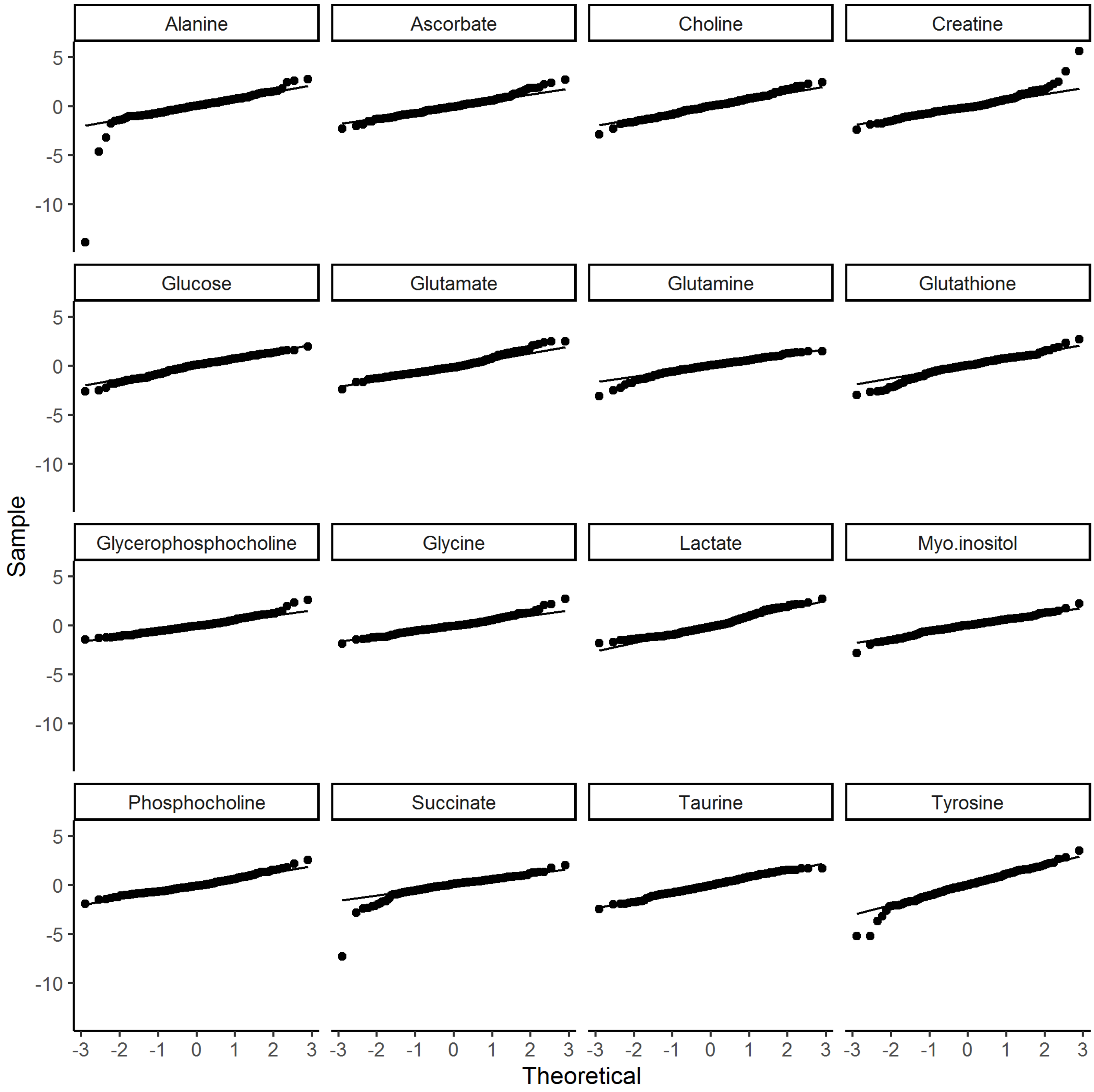

Figure S12. Residuals from the linear mixed models used to assess the effect of time and treatment on the metabolic profile of breast tumor biopsies. The figure was made with `plot(..., type = "residuals")`.

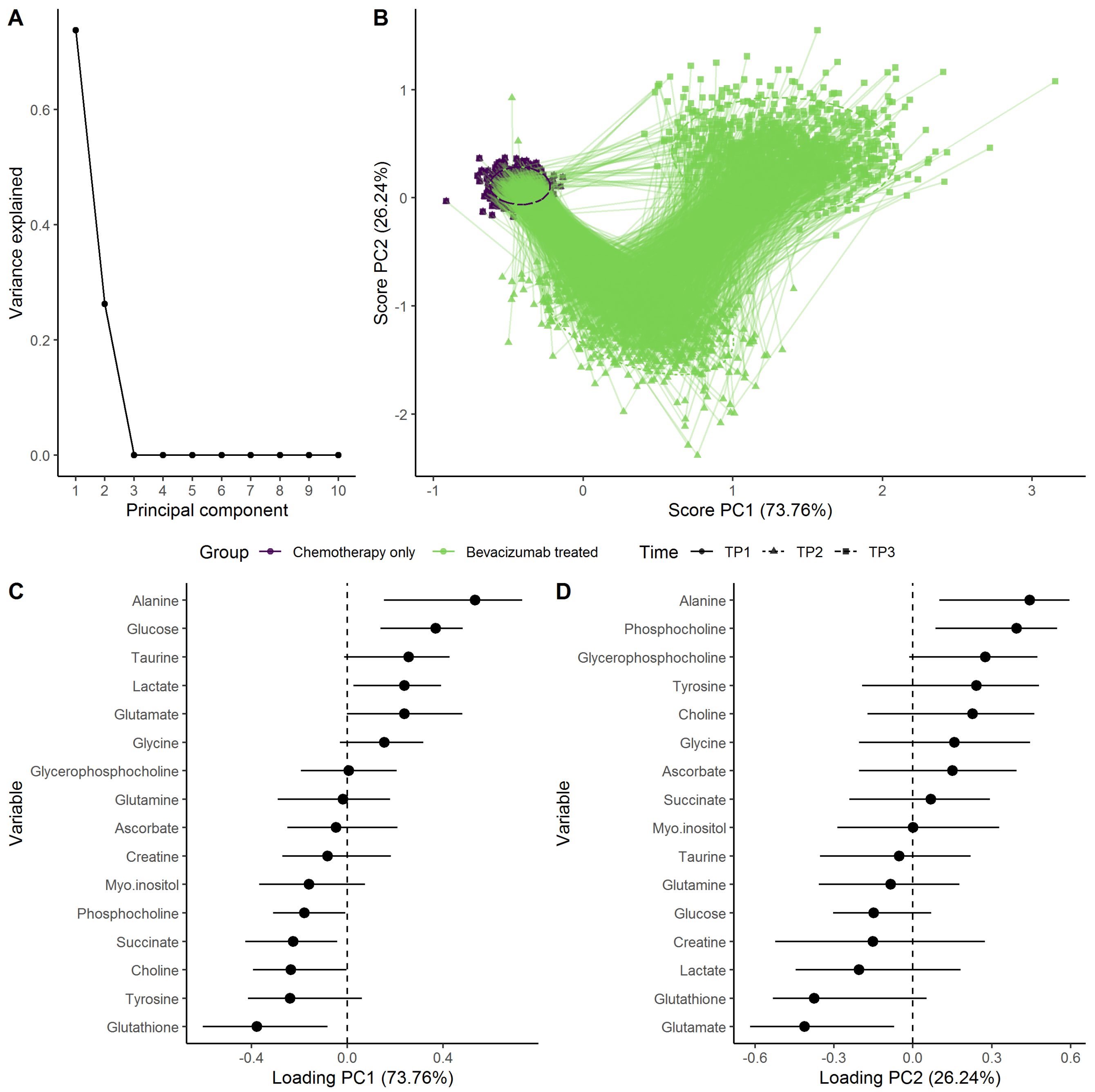

Figure S13. Summary of the main results of an ALASCA model. (**A**) A scree plot demonstrating that most of the variance in the data can be explained by the 1st and 2nd principal components (PC1 and PC2, respectively). (**B**) The scores of PC1 and PC2 calculated for each bootstrap iteration. The time development of the chemotherapy group has been removed so that each bootstrap model is producing a single value for the chemotherapy group that is independent of time. In contrast, most of the bootstrap models shows a characteristic trajectory for the bevacizumab group involving a linear increase in PC1 and a v-shaped development in PC2 with time. (**C**) The loadings of PC1 and (**D**) PC2. The level of metabolites with high loading is increasing when the scores increase and *vice versa*. The plot was made with `plot(..., type = "2D")`.

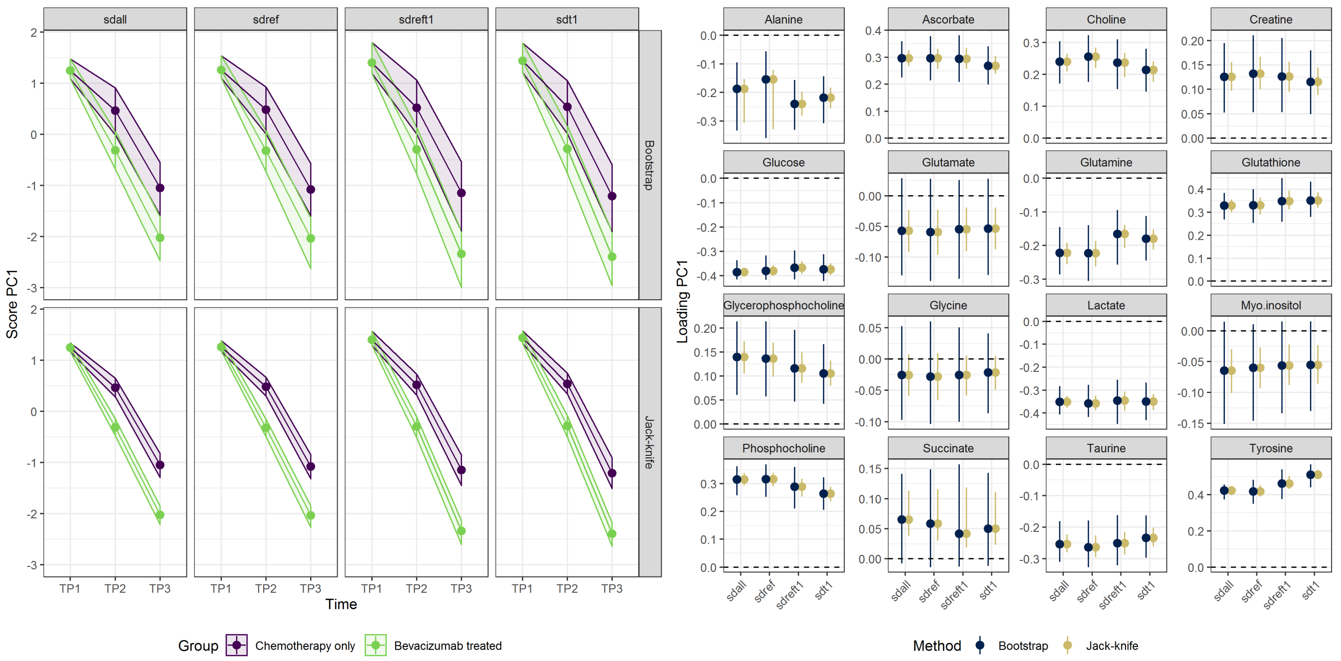

Figure S14. The effect of time and treatment on the metabolic profile of breast tumor biopsies as scores and loadings. Three scaling methods and two resampling strategies are compared.

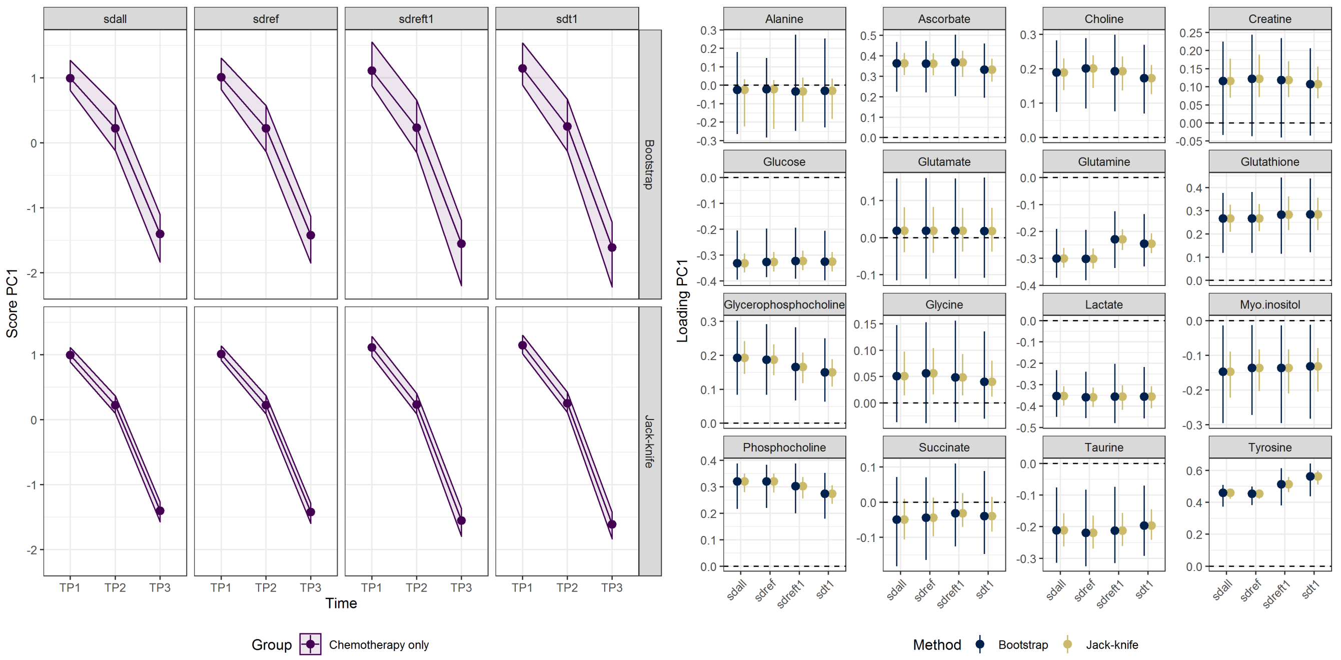

Figure S15. The effect of time and treatment on the metabolic profile of breast tumor biopsies. Three scaling methods and two resampling strategies are compared.

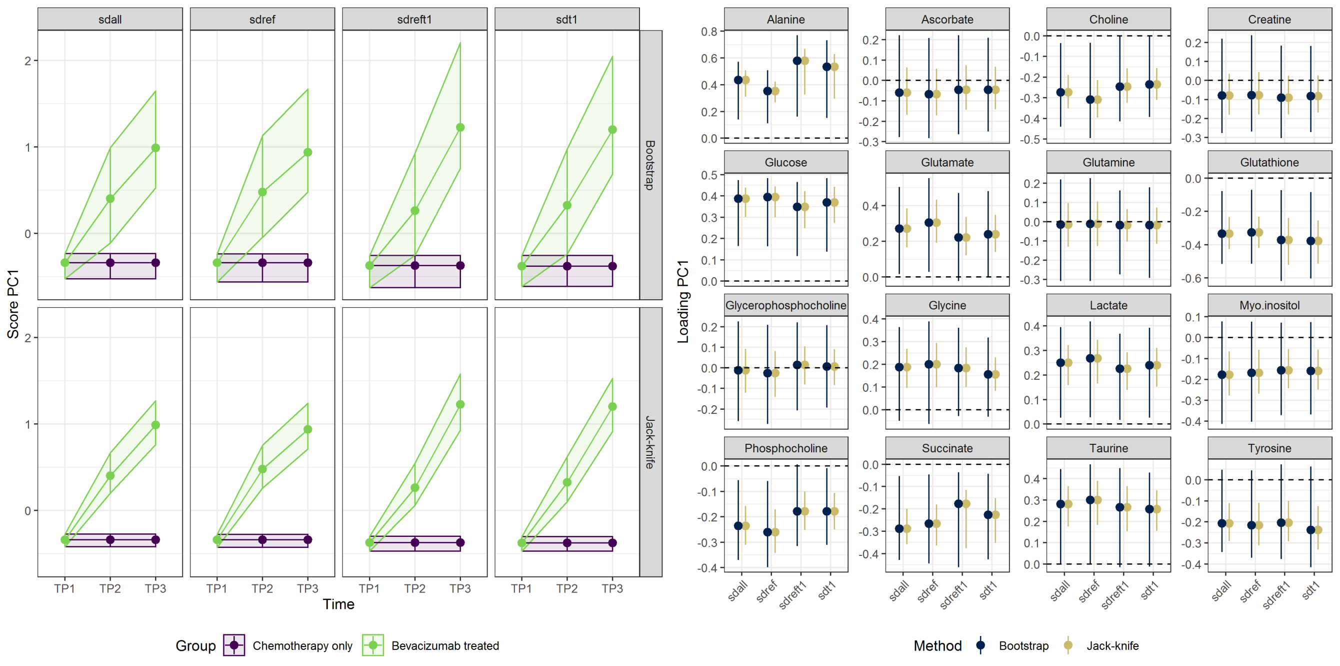

Figure S16. The effect of time and treatment on the metabolic profile of breast tumor biopsies. Three scaling methods and two resampling strategies are compared.

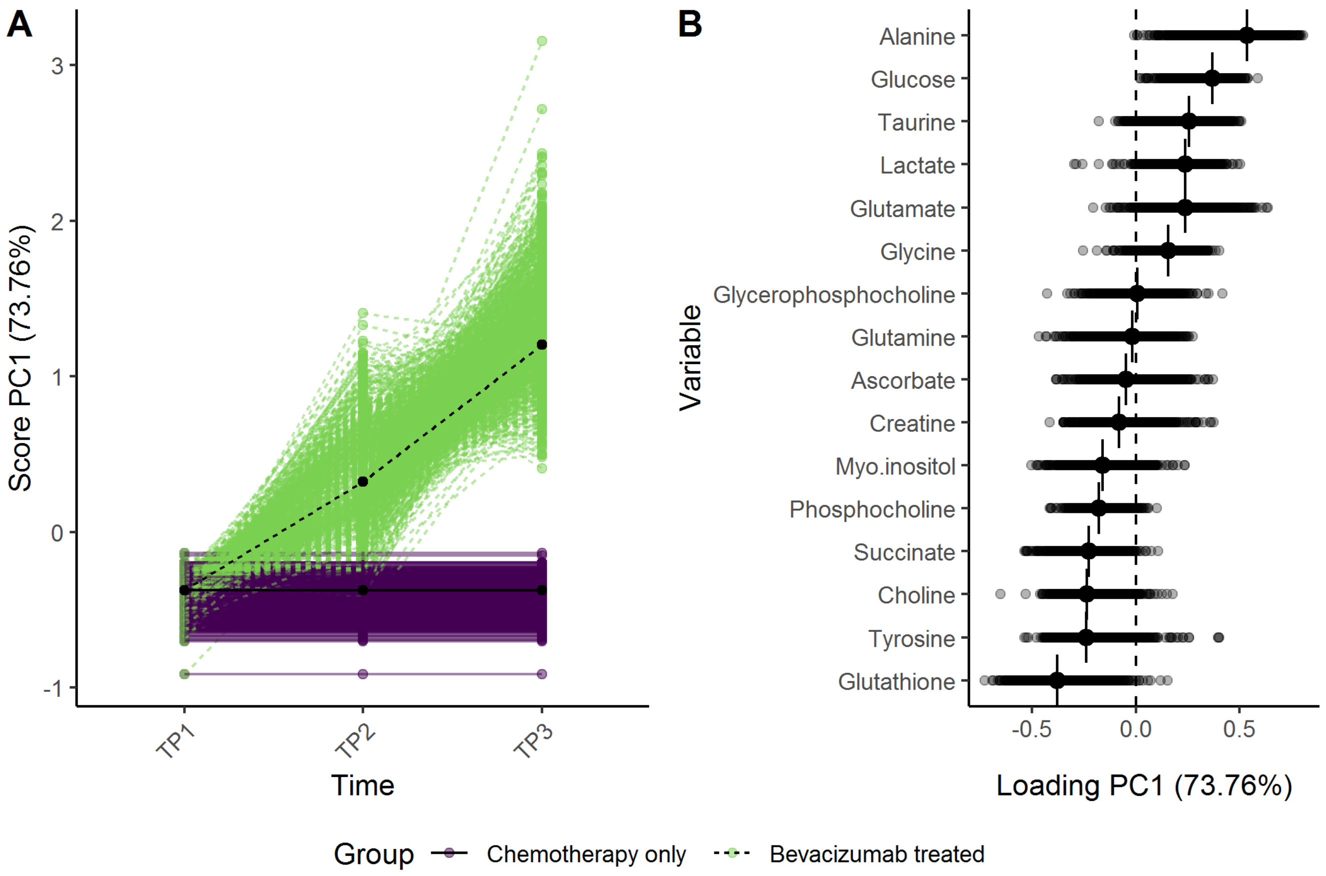

Figure S17. Validation of the RM-ASCA$^+$model shown in Figure 7. The individual bootstrap iterations are shown as colored lines, and the main model is shown with black lines. The plot was made with `plot(..., type = "validation")`.

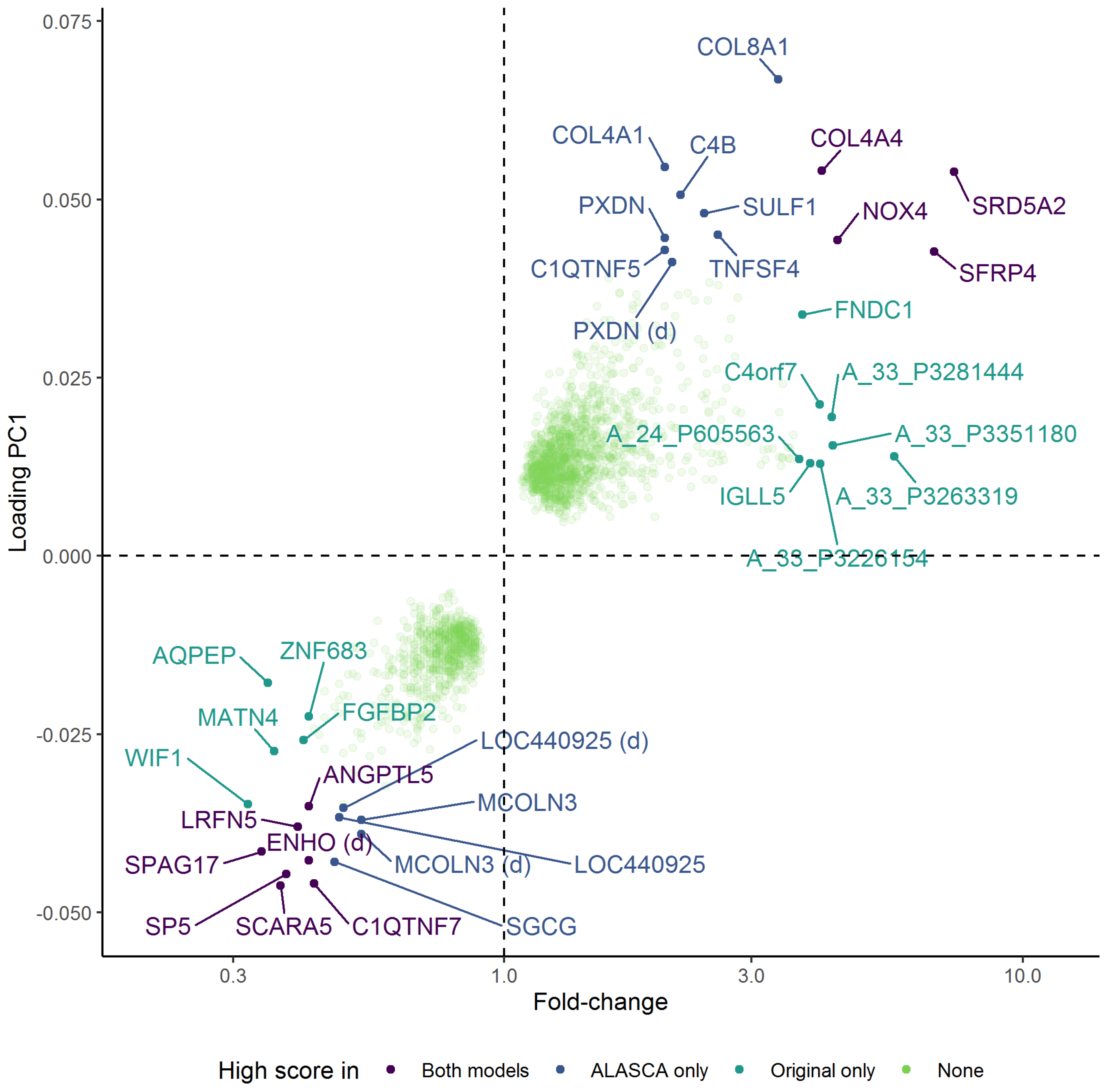

Figure S18. Comparison of RM-ASCA^{+}loadings (Figure 10) to fold-change from the original publication regarding the difference between healthy controls and patients with systemic sclerosis. The 12 genes with expression being strongest affected by systemic sclerosis were selected from both studies.

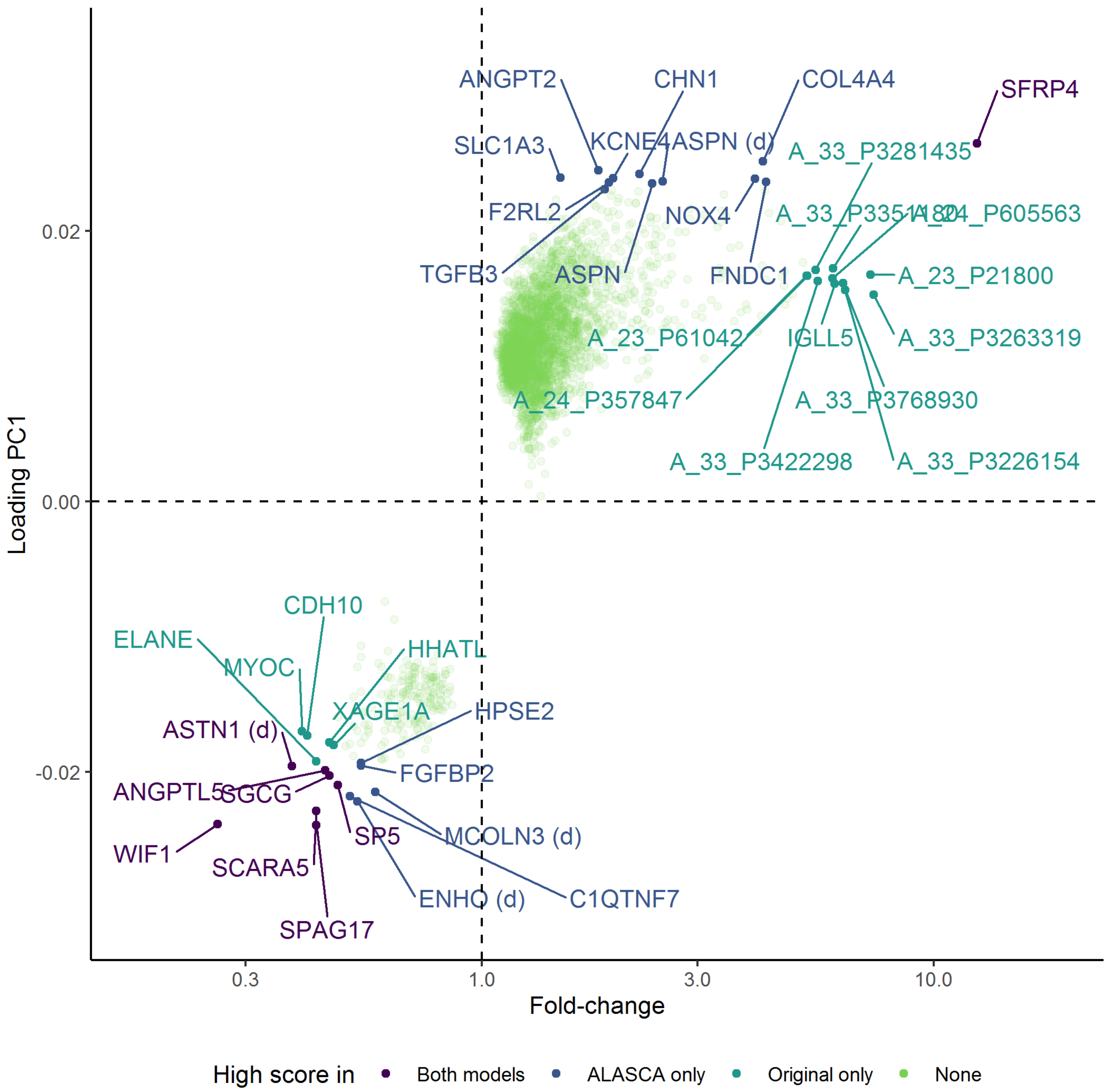

Figure S19. Comparison of RM-ASCA^{+}loadings (Figure 12) to fold-change from the original publication regarding the difference between patients with diffuse or limited systemic sclerosis. The 12 genes with expression being strongest affected by systemic sclerosis variant were selected from both studies.

2 SUPPLEMENTARY TABLES

Table S1. Subset of the design matrix for example 1.1.

(Intercept)	timeEarly 2nd trim	timeLate 2nd trim	timeEarly 3rd trim	timeLate 3rd trim
1	0	0	0	0
1	1	0	0	0
1	0	1	0	0
1	0	0	1	0
1	0	0	0	1

Table S2. Subset of the design matrix for example 1.2.

(Intercept)	timeEarly 2nd trim	timeLate 2nd trim	timeEarly 3rd trim	timeLate 3rd trim	groupYes	BMI	timeEarly 2nd trim:groupYes	timeLate 2nd trim:groupYes	timeEarly 3rd trim:groupYes	timeLate 3rd trim:groupYes
1	0	0	0	0	0	24.6	0	0	0	0
1	1	0	0	0	0	24.6	0	0	0	0
1	0	1	0	0	0	24.6	0	0	0	0
1	0	0	1	0	0	24.6	0	0	0	0
1	0	0	0	0	0	25	0	0	0	0
1	1	0	0	0	0	25	0	0	0	0
1	0	1	0	0	0	25	0	0	0	0
1	0	0	1	0	0	25	0	0	0	0
1	0	0	0	1	0	25	0	0	0	0
1	0	0	0	0	0	36.6	0	0	0	0
1	1	0	0	0	0	36.6	0	0	0	0
1	0	1	0	0	0	36.6	0	0	0	0
1	0	0	1	0	0	36.6	0	0	0	0
1	0	0	0	1	0	36.6	0	0	0	0
1	1	0	0	0	1	28.5	1	0	0	0
1	0	1	0	0	1	28.5	0	1	0	0
1	0	0	1	0	1	28.5	0	0	1	0
1	0	0	0	1	1	28.5	0	0	0	1

Table S3. Subset of the design matrix for example 1.3.

(Intercept)	timeweek 14-21	timeweek 21-28	timeweek 28-32	groupEO-PE	groupLO-PE	timeweek 14-21:groupEO-PE	timeweek 21-28:groupEO-PE	timeweek 28-32:groupEO-PE	timeweek 14-21:groupLO-PE	timeweek 21-28:groupLO-PE	timeweek 28-32:groupLO-PE
1	1	0	0	1	0	1	0	0	0	0	0
1	0	1	0	1	0	0	1	0	0	0	0
1	0	0	0	0	0	0	0	0	0	0	0
1	1	0	0	0	0	0	0	0	0	0	0
1	0	1	0	0	0	0	0	0	0	0	0
1	0	0	1	0	0	0	0	0	0	0	0
1	0	0	0	1	0	0	0	0	0	0	0
1	0	0	1	1	0	0	0	1	0	0	0
1	0	0	0	0	1	0	0	0	0	0	0
1	1	0	0	0	1	0	0	0	1	0	0
1	0	1	0	0	1	0	0	0	0	1	0
1	0	0	1	0	1	0	0	0	0	0	1

Table S4. Subset of the design matrix for example 2.1.

(Intercept)	timeTP2	timeTP3	timeTP2:groupBevacizumab treated	timeTP3:groupBevacizumab treated
1	0	0	0	0
1	1	0	0	0
1	1	0	1	0
1	0	1	0	0
1	0	1	0	1

Table S5. Subset of the design matrix for example 2.2.

(Intercept)	timeTP2	timeTP3	responseResponder	timeTP2:groupBevacizumab treated	timeTP3:groupBevacizumab treated	timeTP2:responseResponder	timeTP3:responseResponder	timeTP2:responseResponder:groupBevacizumab treated	timeTP3:responseResponder:groupBevacizumab treated
1	0	0	1	0	0	0	0	0	0
1	0	0	0	0	0	0	0	0	0
1	1	0	1	0	0	1	0	0	0
1	1	0	0	1	0	0	0	0	0
1	1	0	0	0	0	0	0	0	0
1	1	0	1	1	0	1	0	1	0
1	0	1	1	0	0	0	1	0	0
1	0	1	0	0	1	0	0	0	0
1	0	1	0	0	0	0	0	0	0
1	0	1	1	0	1	0	1	0	1

Table S6. Subset of the design matrix for example 3.1.

(Intercept)	diseaseSSc	genderMale	age
1	1	1	-1.514
1	0	1	-1.305
1	1	1	-1.313
1	0	1	-0.928
1	1	1	-0.765
1	0	1	-0.337
1	1	1	-0.591
1	0	1	-0.075

Table S7. Subset of the design matrix for example 3.2.

(Intercept)	timeTP2	timeTP3	groupDiffuse	timeTP2:groupDiffuse	timeTP3:groupDiffuse
1	0	0	0	0	0
1	1	0	0	0	0
1	0	1	0	0	0
1	0	0	1	0	0
1	1	0	1	1	0
1	0	1	1	0	1
